# An integrated framework for prognosis prediction and drug response modeling in colorectal liver metastasis drug discovery

**DOI:** 10.1186/s12967-024-05127-5

**Published:** 2024-03-30

**Authors:** Xiuman Zhou, Yuzhen Qian, Chen Ling, Zhuoying He, Peishang Shi, Yanfeng Gao, Xinghua Sui

**Affiliations:** 1 School of Pharmaceutical Sciences (Shenzhen), Shenzhen Campus of Sun Yat-sen University, Shenzhen, Guangdong Province 518107 China; 2 School of Life Sciences, Zhengzhou University, Zhengzhou, 450001 China

**Keywords:** Colorectal liver metastasis, Prognostic biomarker, Deep learning, Drug sensitivity

## Abstract

**Background:**

Colorectal cancer (CRC) is the third most prevalent cancer globally, and liver metastasis (CRLM) is the primary cause of death. Hence, it is essential to discover novel prognostic biomarkers and therapeutic drugs for CRLM.

**Methods:**

This study developed two liver metastasis-associated prognostic signatures based on differentially expressed genes (DEGs) in CRLM. Additionally, we employed an interpretable deep learning model utilizing drug sensitivity databases to identify potential therapeutic drugs for high-risk CRLM patients. Subsequently, in vitro and in vivo experiments were performed to verify the efficacy of these compounds.

**Results:**

These two prognostic models exhibited superior performance compared to previously reported ones. Obatoclax, a BCL-2 inhibitor, showed significant differential responses between high and low risk groups classified by prognostic models, and demonstrated remarkable effectiveness in both Transwell assay and CT26 colorectal liver metastasis mouse model.

**Conclusions:**

This study highlights the significance of developing specialized prognostication approaches and investigating effective therapeutic drugs for patients with CRLM. The application of a deep learning drug response model provides a new drug discovery strategy for translational medicine in precision oncology.

**Supplementary Information:**

The online version contains supplementary material available at 10.1186/s12967-024-05127-5.

## Introduction

Colorectal cancer (CRC) ranks third globally in terms of both incidence and death [1]. About 15%$\sim$25% of patients had CRC liver metastasis (CRLM) at the time of diagnosis [2], and another 20%$\sim$25% had unresectable liver metastasis after radical primary resection [3, 4]. Despite the fact that CRLMs are treated aggressively with a combination of surgical resection, chemotherapy, biologic therapy such as antibodies targeting growth factors [5, 6], and immunotherapy for a subgroup patients with microsatellite instability (MSI-H) [7], liver metastasis-targeted therapies are still lacking. To date, therapeutic strategies targeting a single drug target in tumor cells have demonstrated very limited efficacy, and several randomized trials investigating adjuvant chemotherapy have not shown any improvement in overall survival (OS) [8–10]. There is a critical need for prognostic biomarkers to improve risk stratification and facilitate personalized selection of systemic therapies [11].

The advance of high-throughput technology and the accessibility of multi-omics datasets have facilitated the identification of multiple molecular subtypes and biomarkers associated for primary colorectal cancer (CRC). For example, consensus molecular subtypes (CMS), CRC intrinsic subtypes (CIRS) [12] and Immunoscore [13] emphasized distinct molecular features and tumor microenvironment characteristics to improve prognosis prediction and patients stratification [14–16]. Several recent studies have developed prognostic nomograms for CRLM using clinicopathologic data [17, 18]. However, most studies found no correlation between clinical categorization and treatment response. In light of the limited therapy choices for CRLM patients, it is essential to identify biomarkers for predicting cancer susceptibility and to develop new therapeutic targets for individuals with a high metastatic risk.

The utilization of extensive biomedical data as a tool in drug discovery and development has been adopted as an alternative to de novo drug discovery for the identification of novel therapeutic applications of existing drugs [19]. The emergence of extensive high-throughput screening datasets has greatly advanced research in predictive personalized oncology. Various pharmacogenomic datasets, including Cancer Cell Line Encyclopedia (CCLE) [20], Cancer Therapeutics Response Portal (CTRP) [21], Genomics of Drug Sensitivity in Cancer (GDSC) [22] and the Profiling Relative Inhibition Simultaneously in Mixtures (PRISM) Repurposing databases [23] have assessed nearly 5,000 anticancer and non-oncology drugs that have been deemed safe for human use across nearly 1000 cell lines. These databases have facilitated the development of drug response models that utilize molecular profiles to precisely predict drug response in clinical samples [24, 25]. Additionally, various machine learning approaches have been developed for predicting drug response in cancer. For example, the CMap approach constructed a transcriptional signature of disease by comparing the gene expression patterns of diseased and healthy tissue [26]. A negative correlation between the gene expression signature and the drug’s molecular profile suggests the potential for the drug to counteract the gene expression patterns associated with the malignant or high-risk phenotype, which may indicate therapeutic efficacy. P-NET, a biologically informed deep learning network, assessed molecular drivers for therapeutic targeting and categorized prostate cancer patients according to their degree of treatment resistance [27]. Precily utilized a deep neural network framework to predict the response to cancer therapy with pathway activities and drug descriptors [28]. Most current research on drug repurposing and drug response prediction is still in the early stages of concept validation and experiments at the cellular level. However, there is a lack of in vivo experimental verification, which hinders their potential application and translation in clinical settings.

To tackle the aforementioned concerns, we developed prognostic signatures using differentially expressed genes (DEGs) of colorectal cancer (CRC) patients with liver metastases. These signatures aim to forecast the effectiveness of therapeutic interventions and predict prognosis. The prognostic accuracy of these two signatures surpassed that of previously reported signatures in predicting survival outcomes in patients with colorectal cancer (CRC). In addition, we conducted an extensive computational study using drug sensitivity datasets and a deep learning model to predict drug response and screen potential drugs for high-risk colorectal liver metastases (CRLMs). The candidate drugs were assessed using both in vitro and in vivo assays to validate their effectiveness. Obatoclax, a BCL-2 inhibitor, demonstrated significant inhibition of colorectal liver metastases in a CT26 tumor model. Our study not only provides prognostic markers for colorectal liver metastasis (CRLM), but also suggests potential drug candidates that target the associated signaling pathways.

## Materials and methods

### Data source and processing

The colorectal cancer datasets (GSE68468, GSE41568, GSE81558, GSE17536, and GSE39582) were acquired from the Gene Expression Omnibus (GEO) datasets available at https://www.ncbi.nlm.nih.gov/geo/. Table S1 presents the gene expression profiles and clinical information. We included peritumor normal colon (NC), primary tumor (PT), and liver metastasis (LM) samples from GSE68468, GSE41568, and GSE81558 in our analysis as a discovery cohort to identify genes associated with liver metastasis.

The log2-transformed GEO datasets were annotated and quantile normalized. Using the corresponding platform annotation file, the probes were translated into gene symbols. When multiple probes were associated with the same gene symbol, the probe with the highest signal intensity was selected. The R package sva was utilized to eliminate any potential batch effects across multiple experiments. RNA-sequencing (RNA-seq) data of colorectal cancer were obtained from The Cancer Genome Atlas (TCGA-COAD) database at the National Cancer Institute (NCI) Genomic Data Commons (GDC). Survival data for the TCGA-COAD cohort was obtained from the TCGA Pan-Cancer Clinical Data Resource [29]. The TCGA-COAD dataset was utilized as a training cohort to screen for genes associated with prognosis and to develop prognostic signatures. GSE17536 and GSE39582 were retrieved to assess the clinical significance of the prognostic signatures.

We conducted an examination of the gene expression in specific cell clusters within the tumor microenvironment and investigated the impact of the tumor microenvironment on the sensitivity of drugs. This analysis was based on the single-cell RNA sequencing data obtained from GSE225857, which specifically studied liver metastatic colon cancer [30]. In order to validate the expression of signature genes, the original publication annotated a total of 95,445 immune cells, 41,892 tumor cells, and stromal cells from primary colorectal cancer and matched liver metastasis samples. We performed cell type clustering on the dataset GSE225857 using the Seurat package. We processed the aligned all 7 samples and clustered them into 12 groups: B cell, endothelial cell, macrophage, dendritic cell, CD4^+^ T cell, CD8^+^ T cell, fibroblast, monocytes, NK, plasma, mast and tumor cell. The scRNA-seq analyses and visualization were conducted using R (version 4.0.1).

To predict drug sensitivity, we collected expression profile data from human cancer cell lines from the Broad Institute CCLE project (https://portals.broadinstitute.org/ccle/). The half-maximal inhibitory concentration (IC_50_) was employed to evaluate drug sensitivity. The Cancer Therapeutics Response Portal (CTRP), the Genomics of Drug Sensitivity in Cancer (GDSC2) database, and the PRISM Repurposing database were queried for information regarding drug response in human cancer cell lines. The CTRP database provides sensitivity data for 481 compounds across 835 cancer cell lines, while the PRISM database offers sensitivity data for 1448 compounds across 500 cancer cell lines. The IC_50_ values were log-transformed to indicate treatment sensitivity, with lower values indicating greater sensitivity. The k-nearest neighbors (KNN) imputation procedure was used to fill in missing values for drugs marked as not available (NAs), but only for those drugs that had missing values in less than or equal to 20% of the samples.

### Development and validation of the metastasis associated prognostic signatures

Using the R package limma, we identified genes associated with liver metastasis that were differentially expressed in the discovery cohort containing colorectal liver metastases (GSE68468, GSE41568, and GSE81558 after batch effect removal). This analysis compared gene expression profiles of 143 liver metastases, 64 normal colon samples, and 257 primary tumor samples. The differential expression was determined based on a significance threshold of *P* < 0.001 and a fold change greater than 1 or less than − 1. The survival information was not available in the discovery cohort. In order to determine the prognostic significance of 455 DEGs linked to liver metastases in the TCGA-COAD cohort (*n* = 436), we utilized log rank test and univariable Cox proportional hazards regression (*P* < 0.05). Prognostic models were developed using overall survival (OS) and progression-free interval (PFI) as survival endpoints, respectively. Utilizing the LASSO Cox regression model with 1000 iterations and the glmnet function in R, the most informative prognostic markers among the candidate DEGs in the training cohort were identified. The optimal lambda value was determined by 10-fold cross-validation. The optimal prognosis signature was identified by examining the combination of DEGs with the highest concordance index. Based on the selected genes, a multivariate Cox regression risk prediction model was constructed. The liver metastasis associated overall survival signature (MAOS) and metastasis associated progression signature (MAPS) were calculated for each patient by summing the products of all signature genes’ regression coefficients multiplied by their corresponding z-score standardized expression values. Supplementary Table S4 contains the coefficients of glmnet as well as detailed information on MAOS and MAPS signature genes. Supplementary Tables S2 and S3 present the results of univariate Cox models for MAOS and MAPS signature genes in the TCGA-COAD training set.

Then we conducted time-dependent Area Under the Curve (AUC) analyses of Receiver Operating Characteristics (ROC) to assess the predictive performance of MAOS and MAPS in predicting survival outcomes in CRC patients. This analysis was pivotal in determining if MAOS and MAPS could outperform other established prognostic signatures in the context of CRC. The comparison involved external validation datasets GSE39582 and GSE17536, using the MAOS and MAPS signatures against other published signatures: the TME-related gene signature by Zhang et al. [31], the four gene signature by Yuan et al. [32], and the five gene signature by Sun et al. [33]. The true positive rate (sensitivity) and false positive rate (specificity) were computed for each signature at different time intervals. This allowed us to evaluate the probability of predicting survival status at each time point. This procedure generated a sequence of ROC curves, which provided a quantitative assessment of the predictive efficacy of our signatures over time in comparison to previously published signatures.

### Estimating drug response in clinical cohort

The Precily model, a deep neural network (DNN) framework [28], was trained to predict drug responses using high-throughput screening data from cancer cell lines. Precily employs a deep neural network (DNN) architecture with 2–6 hidden layers. DNNs are capable of identifying intricate patterns in large datasets, essential for drug response prediction. The number of layers is adjustable as a hyper-parameter, allowing flexibility and optimization for different datasets and prediction requirements​​. For each of the 550 cancer cell lines with available drug response data in GDSC, pathway enrichment scores were calculated. These scores pertained to 1329 canonical pathways from the Molecular Signatures Database (MSigDB). Numeric molecular descriptors for 173 anti-cancer compounds, as reported in GDSC, were obtained using SMILESVec. This involved supplying the Simplified Molecular-Input Line-Entry System (SMILES) notation, which was retrieved using PubChemPy.

The pathway enrichment scores and drug features were treated as explanatory variables, while the natural log (LN) of half maximal inhibitory concentration (IC_50_) estimates were used as the dependent variable. The deep neural network (DNN) is composed of two layers: an input layer, which contains all 1,429 features, and a fixed hidden layer, which has an activation function of Rectified Linear Unit (RELU) and has a dimension of 512. The first two layers are kept constant. The Keras Tuner library is used with Hyperband and 5-fold cross-validation to optimize hyperparameters. These include the number of layers (2 to 6), the number of neurons (128 to 256), dropout rates (0.1 to 0.5), ADAM optimizer with various learning rates, and Mean Squared Error (MSE) as the loss function.

The model is trained using data from the CCLE/GDSC2 dataset, which includes 80,056 cell line-drug combinations. 173 compounds with SMILES descriptors in the GDSC2 dataset were used to derive these combinations, which involve 550 cell lines from the CCLE dataset. To ensure that there was no overlap in the cell lines, we divided the CCLE/GDSC2 training dataset into a 90% training set and a 10% test set. The DNN model was trained on the entire dataset with fold-specific tuned hyperparameters. Five models were trained for 50 epochs with a batch size of 128 using fold-specific tailored hyperparameters on the whole dataset. After model training, we computed compound descriptors of vector size 100 for 481 CTRP and 4514 PRISM drugs for model predictions.

### Identification of potential therapeutic drugs for high risk cohort

Prior to utilizing the CCLE/GDSC trained Precily model for drug response prediction in the 143 LM samples, we integrated the pathway score matrix with the drug features of vector size 100 for each molecular compound in the CTRP and PRISM databases. 143 samples from colorectal cancer (CRC) patients with liver metastases were divided into low- and high-risk groups based on the median values of MAOS and MAPS scores. We employed Spearman correlation analysis to examine the relationship between IC_50_ value and risk score (MAOS and MAPS). Drugs exhibiting a significant Pearson’s correlation (*P* < 0.05) were chosen. We also considered drugs that showed statistically significant differences (*P* < 0.05, two-sided Wilcoxon rank-sum test) between the highest and lowest quartiles of MAOS and MAPS scores as potential candidates. We also estimated the IC_50_ values of nine approved drugs for CRLM in high and low-risk groups. These drugs include fruquintinib, capecitabine, trifluridine, raltitrexed, regorafenib, mitomycin, fluorouracil, oxaliplatin, and irinotecan.

To explore how the tumor microenvironment influences drug sensitivity, the Precily model was employed to predict IC_50_ values for various approved and candidate drugs in each cell type of the seven CRLM patients from the scRNA-seq dataset GSE225857. We applied Precily to predict IC_50_ values of nine approved and candidate drugs in the seven CRLM patients and investigate the effect of the tumor microenvironment on the drug sensitivity. To evaluate the risk of CRLM patients in the scRNA-seq dataset, we summarized scRNA-seq data into pseudobulk RNA-Seq data. CRLM patients were categorized into high and low risk groups based on the median values of MAOS and MAPS scores, respectively. Subsequently, we compared the pathway enrichment between patients with high and low MAOS/MAPS scores to determine these cell-type-specific pathways. Further, we utilized the Precily model to predict drug sensitivity for each cell type and conducted a screening process to identify therapeutic options that are particularly effective for patients categorized as high-risk based on their MAOS and MAPS scores.

### Cell culture

The murine colorectal cancer cell line CT26 (gifted from Professor Shengdian Wang, Chinese Academy of Sciences) and human HEK-293T cells used for the lentivirus package were cultured with the DMEM medium. The fetal bovine serum (10%), an antibiotic cocktail of penicillin and streptomycin (Sangon Biotech, China), were added to the DMEM medium. All of the cells were cultured in a humidified atmosphere at 37 °C with 5% CO_2_.

### Lentiviral vector constructs and transfection

The pLVX-Luciferase2-P2A-mCherry vector was constructed with the backbone plasmid pLVX-puro (Clontech, 632164) by inserting the genes of firefly luciferase 2 cloned from the commercial vector pGL4.51 (Promega) and mCherry cloned from the vector pLVX-IRES-mCherry (Clontech, 631237). The lentivirus was packaged by HEK-293T cells with pLVX-Luciferase2-P2A-mCherry and the helper plasmids psPAX2 (Addgene) and pMD2.G (Addgene). The CT26-Luc2 cell line was established by transfecting it with the lentivirus, and the efficiency was verified by detecting the fluorescence of mCherry.

### MTT and transwell assays

Cell cytotoxicity of the compounds was tested by the MTT assay. The CRC cell line CT26 was seeded in 96-well plates with a concentration of 4 × 10^3^ cell/well, and exposed to compounds with a series of diluted concentrations from 30 µM to 0.3 µM. After being treated for 24, 48 and 72 h, 20 µL MTT (5 mg/mL) was added to label the live cells and incubated at 37 °C for 4 h. Next, 150 µL DMSO was added to dissolve the formazan crystals for 10 min. Absorbance values were measured at 490 nm and 570 nm (for Obatoclax). The MTT assay for each compound was repeated at least three times. The Transwell migration assay was performed with cells seeded in chambers in 24-well plates. The 4 × 10^4^ cells/well CT26 cells were seeded into the upper chamber (pore size, 8 μm), with the well supplemented with 600 µL of DMEM medieum and exposed to the indicated concentrations of compounds at a concentration of 10 µM or 30 µM (TargetMol, USA). After a 24 h incubation, cells were fixed with 4% paraformaldehyde (Solarbio, #P1110, Beijing, China), stained with 0.2% crystal violet (Solarbio, #G1062), and then photographed.

### The colorectal liver metastasis model and IVIS imaging

Female BALB/c mice of 18–20 g (age of 6–7 weeks) from Vital River Laboratories were used for determining the anti-tumor and metastasis effects. The experiments were performed in accordance with the protocol and the approval of the Ethics Committee of Zhengzhou University. The colorectal liver metastasis model was established by the injection of 5 × 10^5^ CT26-Luc2 cells into the spleen, followed by splenectomy as previously reported [34]. The spleens of BALB/c mice were surgically exposed and injected with CT26-Luc2 cells in 50 µL of sterile PBS (*n* = 6 per group). The tumor growth and metastasis were detected by tracking the luciferase signal with the IVIS Lumina (PerkinElmer, USA). Mice were divided into three groups according to the luciferase signal acquired on the 3rd day post tumor inoculation by random, which is regarded as day 0 for following treatment. The mice were given daily intravenous injections of normal saline, 2 mg/kg or 5 mg/kg Obatoclax (TargetMol, USA) in 1% Tween-80 and 1.5% DMSO. The luciferase signals were tracked 7 or 13 days later. Upon the completion of the treatment, the mice were euthanized, and the livers were immediately removed, tracked with the luciferase signals, and weighted.

### Statistical analysis

The R program (version 4.0.1) was utilized for statistical analysis and visualization. We used the R packages survminer and survivor to perform univariate Cox regression analysis. Survival analysis was performed utilizing Kaplan-Meier (KM) methods, and the log-rank test was used to assess statistical significance. The timeROC R package was used to calculate the time-dependent area under the receiver operating characteristic curve (AUC). A two-tailed P value of less than 0.05 was deemed statistically significant for all statistical analyses. Pathway analyses were conducted on the 50 hallmark pathways and 1329 canonical pathways from the Molecular Signature Database. This was done using the GSEABase and GSVA packages to perform Gene Set Variation Analysis (GSVA) [35].

## Results

### Identification of liver metastasis related DEGs

**Fig. 1:**
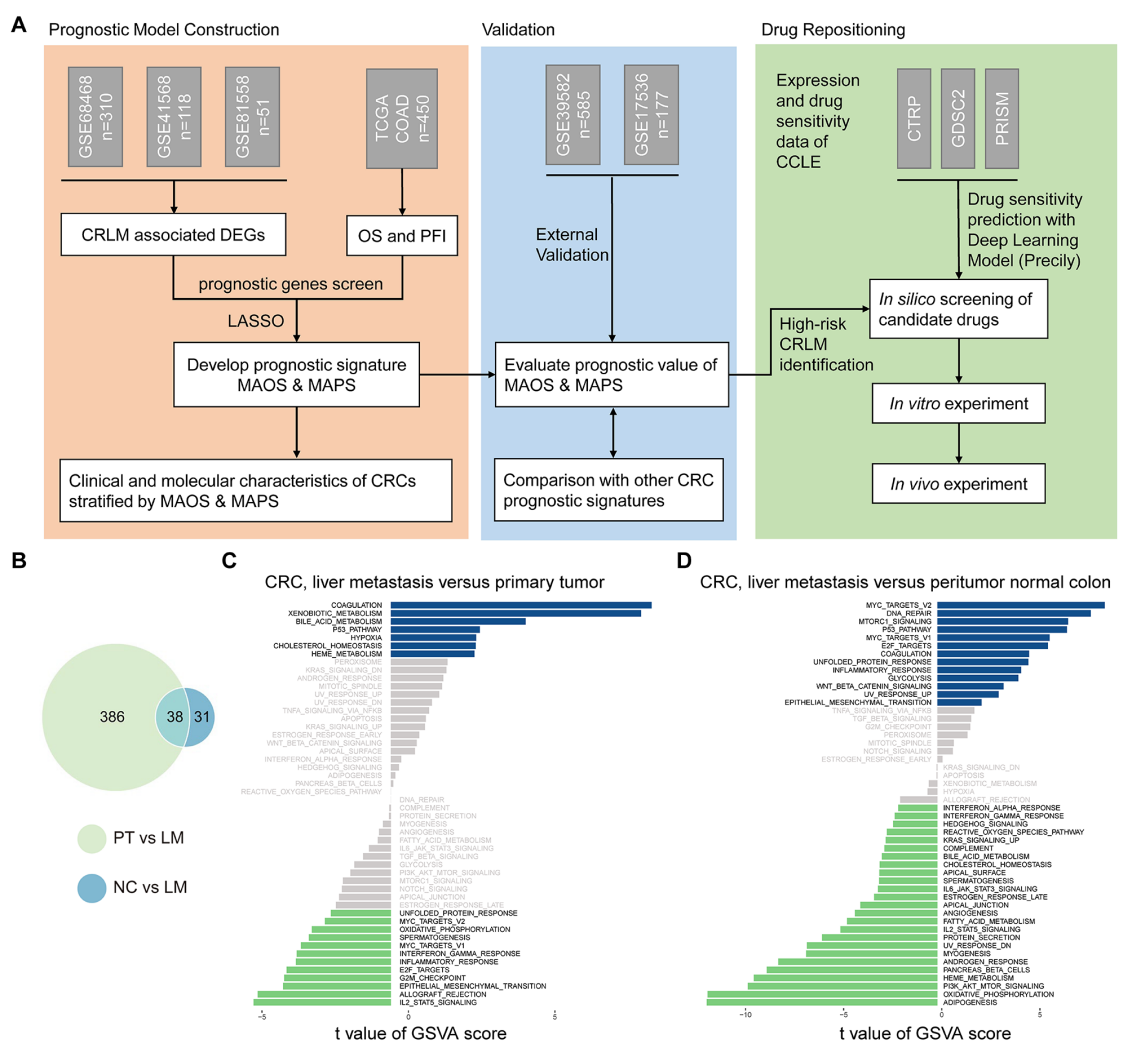
Differentially expressed genes and enriched pathways associated with liver metastases. (**A**) Schematic diagram of the study design. (**B**) Venn diagram depicting common liver metastasis-related genes shared by NC vs. LM group and PT vs. LM group. (**C**) Differences in pathway activities scored by GSVA between liver metastasis tumor and peritumor normal colon. (**D**) Differences in pathway activities scored by GSVA between liver metastasis tumor and primary colon tumor. NC: peritumor normal colon; LM, liver metastasis; PT, primary colorectal tumor

A systematical flow chart was illustrated in Fig. 1A. Considering the limited number of surgical biopsies of liver metastasis, we combined a cohort of 462 samples from GSE68468, GSE41568 and GSE81558, including 63 peritumor normal colon (NC) samples, 256 primary tumor (PT) samples and 143 liver metastasis (LM) samples after removing potential batch effects (Supplementary Figure S1). A total of 455 differentially expressed genes (DEGs) were identified as associated with liver metastasis in colorectal cancer. The Venn diagram illustrated 38 common DEGs between the LM vs. NC group and the PT vs. LM group (Fig. 1B). 424 DEGs were identified in liver metastasis samples compared with peritumor normal colon samples (LM vs. NC group), of which 164 were up-regulated and 260 down-regulated. 69 DEGs were identified in liver metastasis samples compared with primary tumor samples (LM vs. PT group), of which 54 were up-regulated and 15 down-regulated. To investigate the molecular processes underlying the process of colorectal liver metastasis, we performed GSVA and KEGG pathway enrichment analysis using cancer hallmark and KEGG pathway gene sets from the Molecular Signatures Database v7.4. GSEA analysis of hallmark gene sets revealed that the up-regulated genes in liver metastasis were significantly enriched in multiple pathways associated with carcinogenesis, including coagulation, P53, and hypoxia, as well as metabolic pathways related to xenobiotic and bile acid metabolism, in comparison to the primary tumor (Fig. 1C). In contrast, liver metastasis showed a decrease in gene expression related to processes such as proliferation (e.g., Myc targets v1 and Myc targets v2) and epithelial mesenchymal transition (EMT). Additionally, immune activation pathways, including the interferon-γ response and inflammatory response, were also down-regulated in liver metastasis. In comparison to peritumor normal colon, certain pathways showed up-regulation, including DNA repair, MYC Targets v1 and v2, MTORC1 signaling, and E2F targets. Conversely, metabolic pathways such as adipogenesis, oxidative phosphorylation, fatty acid metabolism, and bile acid metabolism exhibited down-regulation (Fig. 1D).

### Construction of liver metastasis related prognostic signatures

We conducted univariate Cox regression analysis and Kaplan-Meier (KM) analysis to further evaluate the prognostic value of DEGs associated with liver metastasis. We developed two liver metastasis-associated signatures, liver metastasis associated overall survival signature (MAOS) and metastasis associated progression signature (MAPS), using the TCGA COAD cohort. These signatures were constructed based on overall survival (OS) and progression-free interval (PFI) as survival outcomes. The MAOS signature consisted of 10 genes: ATOH1, CXCL1, FABP4, INHBB, LGALS4, MEGF6, NAT1, SCGB2A1, and SERPINA1. The MAPS signature comprised 11 genes, namely CFHR4, CXCL11, F5, INHBB, LGALS4, MEGF6, NAT1, S100A2, SERPINE1, SRPX, and VEGFA. The genes INHBB, LGALS4, MEGF6, and NAT1 were found to be common in both signatures. CXCL1 and CXCL11 are chemokine ligands that exhibit a positive correlation. Both SERPINA1 and SERPINE1 are members of the Serpin Family. The KM survival curve for each gene was shown in Supplementary Figure S2 and S3.

**Fig. 2:**
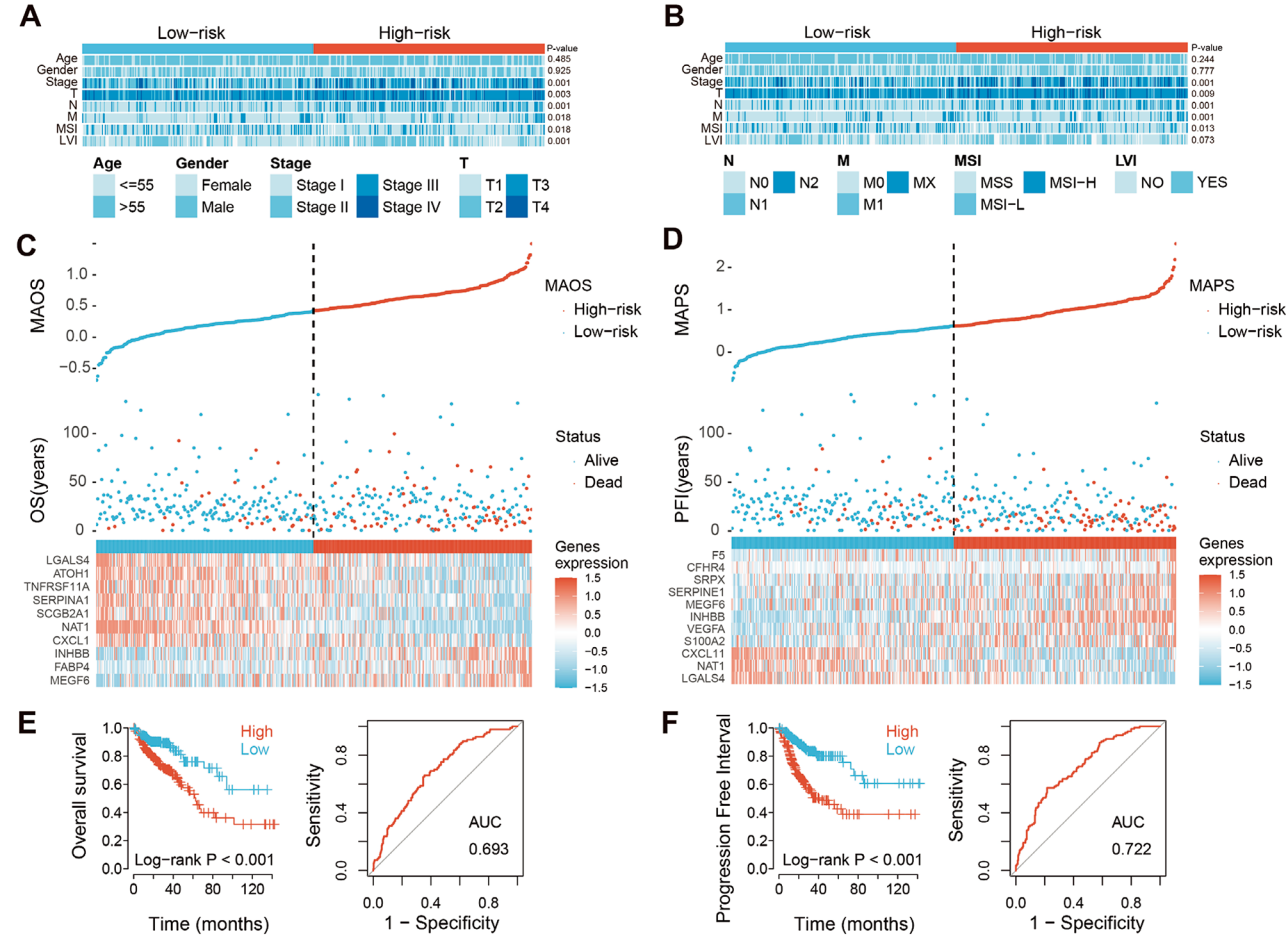
Construction of MAOS and MAPS signatures in TCGA training set. (**A** and **B**) Clinical characteristics of MAOS and MAPS signatures in TCGA COAD cohorts, respectively. T, tumor size and local growth; N, extent of lymph node metastases; M, occurrence of distant metastases in tumor-node-metastasis (TNM) system. MSI, microsatellite instability; LVI, Lymphovascular invasion. (**C** and **D**) The distribution of risk score, survival status and gene expression panel in the TCGA training set for MAOS and MAPS, respectively. For each patient, a risk score was calculated based on the prognostic signature, and all patients are displayed (sorted from low to high score). The vertical dotted line indicates the median cutoff dividing patients into low-risk and high-risk groups. (**E** and **F**) Kaplan-Meier survival analysis and ROC curve analysis for patients in TCGA training set of MAOS and MAPS, respectively

We subsequently investigated the correlation between MAOS/MAPS scores and clinical characteristics. The findings indicated a significantly correlation between MAOS and MAPS with high TNM stages (*P* = 0.001), lymph node metastases (N) (*P* = 0.001), tumor size (T) (MAOS: *P* = 0.003 and MAPS: *P* = 0.009), occurrence of distant metastases (M) (MAOS: *P* = 0.018 and MAPS: *P* = 0.001) and microsatellite instability (MAOS: *P* = 0.018 and MAP: *P* = 0.013) (Fig. 2A and B). Furthermore, there was a significant correlation between lymph vascular invasion (LVI) and metastasis-associated overall survival (MAOS), but no significant correlation was observed between LVI and metastasis-associated progression-free survival (MAPS).

Patients in the training and testing sets were classified as high or low risk in the Kaplan-Meier survival analysis, according to the median values of MAOS and MAPS, respectively. Figure 2C and D depict the survival status, MAOS/MAPS scores, and signature gene expression of colorectal cancer (CRC) patients in the training group. The median survival time of patients in the low-risk group for MAOS or MAPS in the TCGA COAD cohort was considerably longer than that of patients in the high-risk group (*P* < 0.001, Fig. 2E and F). The prognostic model for overall survival (OS) achieved an area under the curve (AUC) of 0.693, while the model for progression-free interval (PFI) achieved an AUC of 0.722.

### MAOS and MAPS are independent of conventional CRC clinical characteristics

**Fig. 3:**
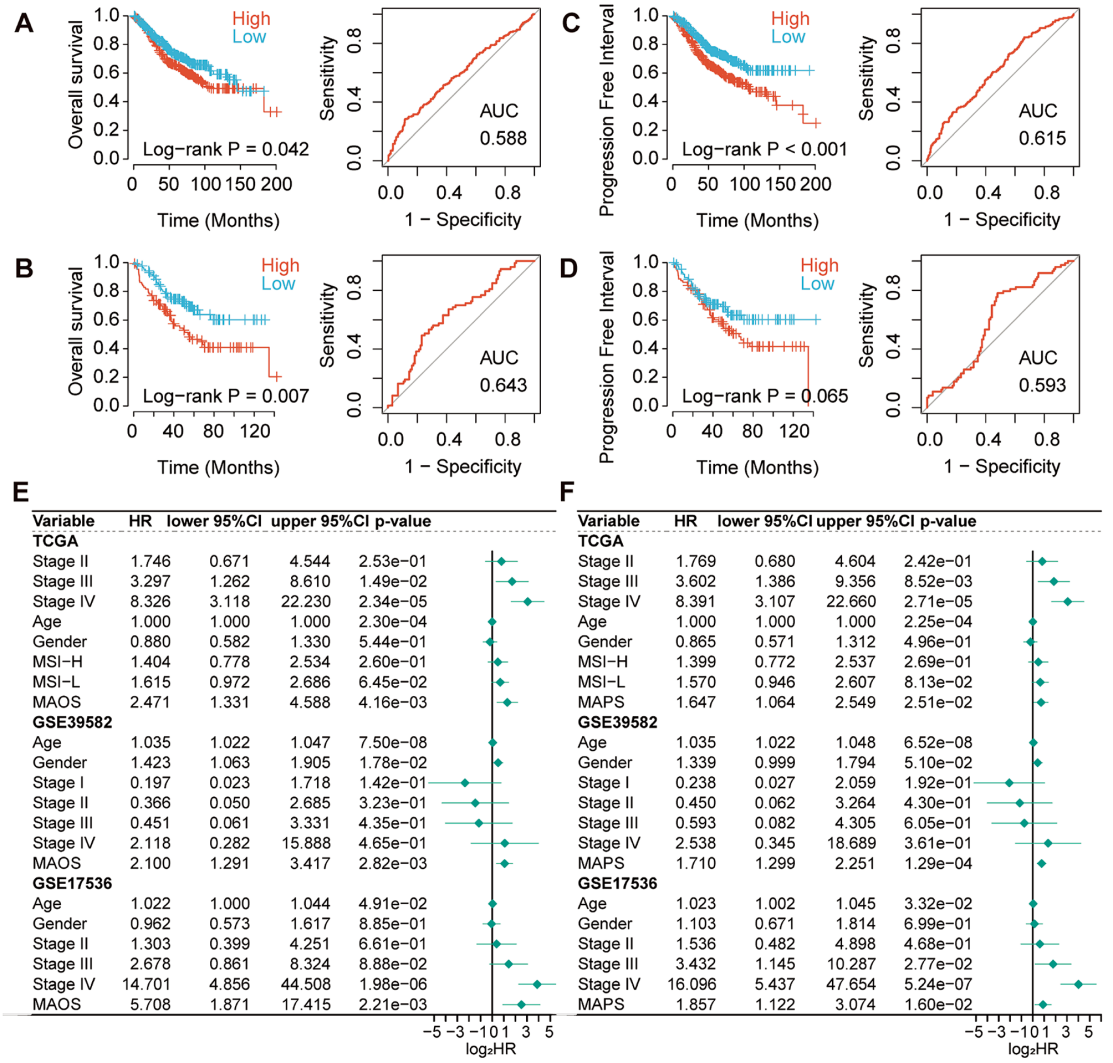
Validation of MAOS and MAPS signatures in training and validation datasets. (**A** and **B**) Kaplan-Meier plots and the receiver operating characteristic (ROC) curve of MAOS in GSE39582 and GSE17536 validation sets. (**C** and **D**) Kaplan-Meier plots and ROC curve of MAPS in the two validation datasets (GSE39582 and GSE17536). (**E** and **F**) The multivariate Cox analysis of the MAOS and MAPS signature with other clinicopathological factors in the training TYGA-COAD datasets and two validation datasets (GSE39582 and GSE17536), respectively

Before validating the prognostic signatures MAOS and MAPS with independent datasets, we analyzed several typical clinical characteristics with the signatures in the TCGA training set. Table 1 demonstrates significant differences in conventional clinical pathological factors, including microsatellite status, T, N, M stage, and lymphatic invasion status, between high and low risk groups based on MAPS and MAOS risk signatures. More than 60% of patients in stage III and IV were classified as high-risk based on either MAPS or MAOS. In the GSE39582 dataset, patients classified as low-risk for MAOS exhibited a notably extended overall survival (OS) in comparison to the high-risk group (*P* = 0.042, AUC = 0.588, Fig. 3A). In the GSE17536 dataset, individuals classified as low-risk for MAOS also exhibited a considerably longer overall survival (OS) compared to those classified as high-risk (*P* = 0.007, AUC = 0.643, Fig. 3B). Patients in the MAPS low-risk group of the GSE39582 dataset exhibited a significantly longer PFI (*P* < 0.001, AUC = 0.615, Fig. 3C). However, in the GSE17536 dataset, the difference in PFI was not significant (*P* = 0.065, AUC = 0.593, Fig. 3D), likely due to the smaller sample size of GSE17536.

**Table 1:** Clinical characteristics of high- and low- risk groups according to median values of MAPS and MAOS in the TCGA COAD dataset

	MAPS		P*	MAOS		P
	High-risk n (%)	Low-risk n (%)		High-risk n (%)	Low-risk n (%)	
n	220	216		219	217	
Microsatellite (%)			0.01			0.008
MSI-H	26 (11.8)	52 (24.1)		26 (11.9)	52 (24.0)	
MSI-L	39 (17.7)	34 (15.7)		36 (16.4)	37 (17.1)	
MSS	150 (68.2)	127 (58.8)		153 (69.9)	124 (57.1)	
Gender = male (%)	118 (53.6)	112 (51.9)	0.782	116 (53.0)	114 (52.5)	1
T (%)			0.011			0.005
T1	3 (1.4)	7 (3.2)		3 (1.4)	7 (3.2)	
T2	30 (13.6)	44 (20.4)		27 (12.3)	47 (21.7)	
T3	151 (68.6)	148 (68.5)		154 (70.3)	145 (66.8)	
T4	36 (16.4)	17 (7.9)		35 (16.0)	18 (8.3)	
N (%)			< 0.001			< 0.001
N0	104 (47.3)	151 (69.9)		100 (45.7)	155 (71.4)	
N1	57 (25.9)	44 (20.4)		56 (25.6)	45 (20.7)	
N2	59 (26.8)	21 (9.7)		63 (28.8)	17 (7.8)	
M (%)			< 0.001			0.022
M0	147 (66.8)	180 (83.3)		154 (70.3)	173 (79.7)	
M1	47 (21.4)	16 (7.4)		43 (19.6)	20 (9.2)	
MX	22 (10.0)	20 (9.3)		20 (9.1)	22 (10.1)	
Stage (%)			< 0.001			< 0.001
Stage I	28 (12.7)	46 (21.3)		26 (11.9)	48 (22.1)	
Stage II	71 (32.3)	102 (47.2)		68 (31.1)	105 (48.4)	
Stage III	74 (33.6)	52 (24.1)		82 (37.4)	44 (20.3)	
Stage IV	47 (21.4)	16 (7.4)		43 (19.6)	20 (9.2)	
LVI (%)			0.105			0.001
No	108 (49.1)	127 (58.8)		98 (44.7)	137 (63.1)	
Yes	92 (41.8)	70 (32.4)		98 (44.7)	64 (29.5)	

MSI, microsatellite instability; LVI, Lymphovascular invasion

*Chi-square test

We conducted multivariate Cox regression analysis on two independent validation sets, GSE39582 and GSE17536, to assess the independence of MAOS and MAPS from common clinical characteristics in predicting CRC prognosis. The multivariate Cox regression analysis revealed that the MAOS and MAPS were independent risk factors for overall survival (OS) in addition to Stage III and IV in the TCGA COAD dataset (HR = 2.47, 95% CI = 1.33–4.59; HR = 1.65, 95% CI = 1.06–2.55). The multivariate Cox analysis in both the GSE39582 and GSE17536 datasets consistently showed significant results for MAOS and MAPS (Fig. 3E and F).

### Evaluation the prognostic value of MAOS and MAPS

**Fig. 4:**
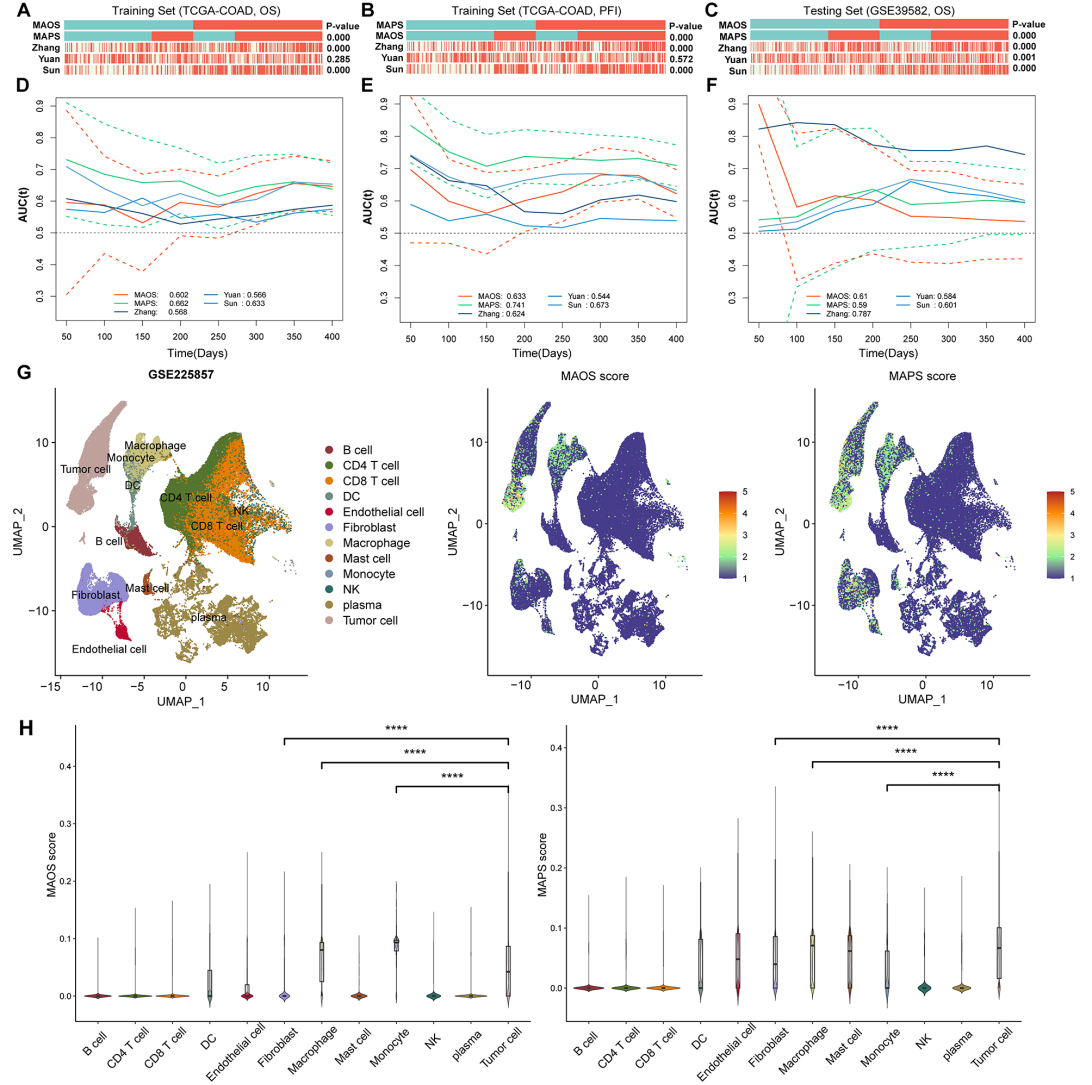
Evaluation of prognostic value and targeting cell types of MAOS and MAPS. (**A**) The association between MAOS, MAPS and other three prognostic signatures in TCGA COAD training dataset using overall survival (OS) information. (**B**) The association between MAOS, MAPS and other three prognostic signatures in TCGA COAD training dataset using progression free interval (PFI) information. (**C**) The association between MAOS, MAPS and other three prognostic signatures in validation dataset using OS information. (**D**) The time-dependent area under the receiver operating characteristic (ROC) curves of MAOS, MAPS and other three prognostic signatures in TCGA COAD training dataset using OS information. (**E**) The time-dependent ROC curves of MAOS, MAPS and other three prognostic signatures in TCGA COAD training dataset using PFI information. (**F**) The time-dependent ROC curves of MAOS, MAPS and other three prognostic signatures in validation dataset using OS information. Survival difference was compared using log-rank test. Red and Green dotted lines on the time-dependent area under the ROC curve plots represent 95% CI of MAOS and MAPS, respectively. (**G**) (left) UMAP plot visualization of all cell subtypes from six CRLM patients. Different cell subtypes were annotated by Seurat algorithm. (middle) UMAP plot visualization of the distribution of MAOS score. (right) UMAP plot visualization of the distribution of MAPS score. (**H**) Violin plot of MAOS (left) and MAPS (right) scores in different cell types. ****, *P* < 0.0001

The prognostic signatures of MAOS and MAPS were compared to formerly published gene signatures. The correlation between MAOS and MAPS with Zhang’s and Sun’s signatures in the TCGA COAD training set was statistically significant (Pearson’s Correlation test, *P* < 0.001). However, there was no significant correlation observed with Yuan’s signature (Fig. 4A and B). Both MAOS and MAPS showed significant correlations with the three signatures in the GSE39582 testing set, indicating a high level of consistency between the training and testing sets (Fig. 4C).

We conducted time-dependent receiver operating characteristic (ROC) analyses to assess the potential of our signatures to enhance the prognostic accuracy for predicting the survival of CRC patients, in comparison to other signatures. The findings indicate that MAPS and MAOS demonstrated superior performance compared to the remaining three signatures in the training set. The MAPS demonstrated superior performance in predicting overall survival (OS) and progression-free interval (PFI) in CRC patients, as shown in Fig. 4D and E. It achieved the highest average area under the curve (AUC) values of 0.662 and 0.741 for OS and PFI, respectively. Zhang’s signature exhibited superior performance in the GSE39582 testing set due to its utilization of GSE39582 as a training set (Fig. 4F). Both MAOS and MAPS demonstrate similar performance when compared to the other two signatures in the testing set.

Additionally, we examined the specific distribution of MAOS and MAPS in CRLM patients using single-cell RNA transcriptome data (GSE225857). We annotated the major cell types and visualized the distribution of MAOS and MAPS scores (Fig. 4G). Violin plot illustrated the elevated expression of the majority of MAOS and MAPS signature genes in tumor cells and myeloid cells, in comparison to other cell types (Fig. 4H). The gene LGALS4 exhibited the highest expression in tumor cells, while the gene VEGFA from the MAPS signature was highly expressed in various cell types, including myeloid cells and tumor cells (Supplementary Figure S4).

### Discovery of candidate drugs for CRLM

**Fig. 5:**
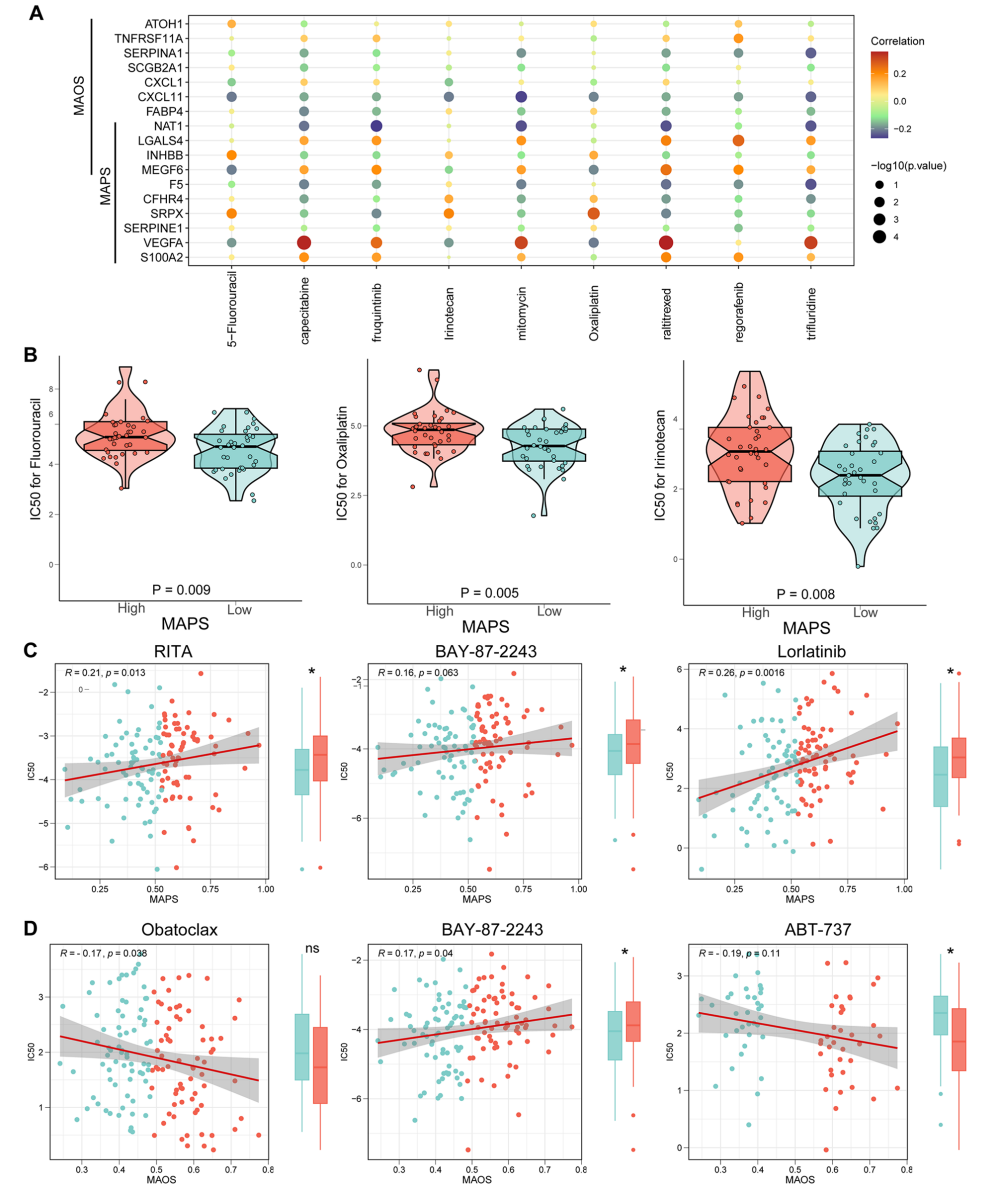
Identification of candidate drugs for CRLM patients. (**A**) Bubble plot of the relationship between approved CRLM drugs and signature genes of MAOS and MAPS. (**B**) Box plots of the comparison of predicted IC_50_ of approved CRLM drugs Fluorouracil, Oxaliplatin and Irinotecan between high- and low-MAPS groups. (**C**) Correlation between the predicted IC_50_ of candidate drugs (RITA, BAY-87-2243 and lorlatinib) and MAPS scores in CRLM cohort. (**D**) Correlation between the predicted IC_50_ of candidate drugs (Obatoclax, BAY-87-2243 and ABT-737) and MAOS scores in CRLM cohort. Lower IC_50_ values imply greater drug sensitivity. *P*-values of boxplots and violin plots were obtained from the two-sided Wilcoxon rank-sum test

We initially determined the IC_50_ values of nine approved drugs for CRLM to assess the efficacy of MAOS and MAPS in predicting drug sensitivity. Figure 5A and B illustrated the correlation and significance between drug sensitivities and signature genes of MAOS and MAPS. The IC_50_ values of fluorouracil, oxaliplatin, and irinotecan were found to be higher in the high-MAPS group. The findings indicated that CRLM patients exhibiting high MAPS scores demonstrated resistance to conventional chemotherapy treatments.

We further screened potential drugs in CTRP and PRISM databases to identify suitable candidates for high-risk CRLM patients. We conducted a drug response prediction to identify drugs that exhibit significant differential responses between high and low risk groups classified by MAOS and MAPS. The results revealed that low MAPS groups exhibited significant sensitivity to one CTRP drug (RITA), and two drugs derived from PRISM (BAY-87-2243 and lorlatinib) (Fig. 5C). Low MAOS groups were also sensitive to BAY-87-2243. There was a notable negative correlation between the MAOS scores and the predicted IC_50_ value of the CTRP drug Obatoclax. ABT-737, another apoptosis-related inhibitor, exhibited statistically significant differences between the highest and lowest quartiles of MAOS scores (Fig. 5D). Obatoclax and ABT-737 may have therapeutic potential for treating chemotherapy-resistant high MAOS CRLM patients, as lower IC_50_ values indicate increased drug sensitivity.

Considerable tumor heterogeneity was identified in the composition of the tumor microenvironment of the CRLM patients in the scRNA-seq dataset (Figure S5A). The drug sensitivity also differed among the CRLM patients, showing the sensitivity of Precily in personalized drug prediction (Figure S5B). We observed considerable variation in predicting drug sensitivity across different cell types within the tumor microenvironment, with macrophages exhibiting the highest degree of variability (Figure S5C). By comparing the pathway enrichment scores between patients with high- and low-MAOS scores (Figure S6), we found significant pathways were enriched in tumor cell, endothelial cell, and fibroblasts. On the other hand, significant pathways were enriched in dendritic cell and CD8^+^ T cells between patients with high- and low-MAPS scores (Figure S7). Interestingly, the cell-type-specific drug screening process confirmed obatoclax and ABT-737 as candidate drugs but targeting different cell types in the MAOS and MAPS risk groups (Figure S8).

### Further identification of the therapeutic drugs

**Fig. 6:**
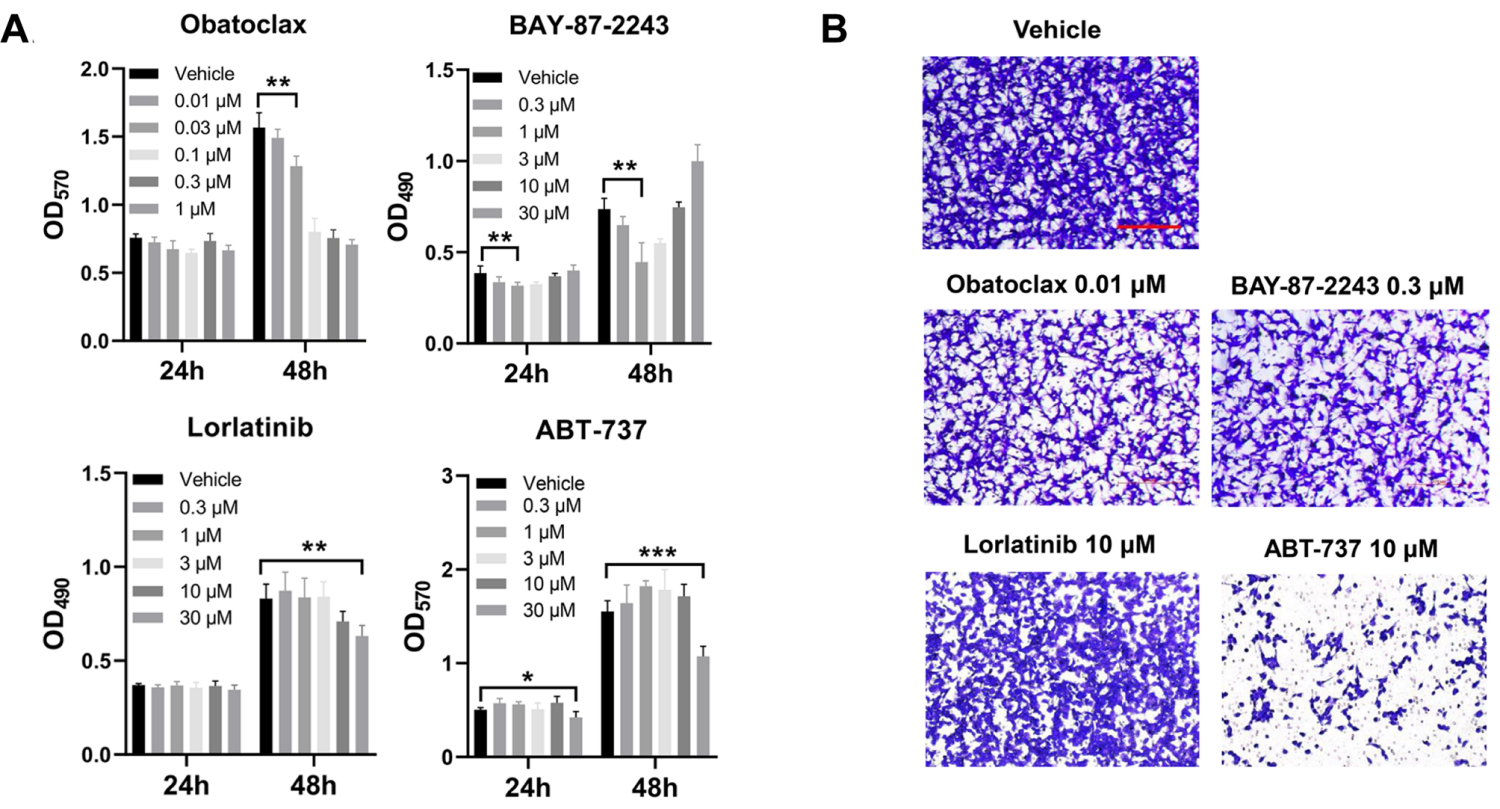
The effects of candidate drugs on the proliferation and migration of CT26 cells. (**A**) Cell proliferation of CT26 cells treated with the candidate drugs at the indicated concentration or the vehicle detected by MTT measuring the absorbance at 490–570 nm. *, *P* < 0.05, **, *P* < 0.01, ***, *P* < 0.001. (**B**) The migration of CT26 cells treated with the candidate drugs at the indicated concentration or the vehicle for 24 h detected by the Transwell assay. Bar = 250 μm. Representative results of at least three independent experiments were shown

To validate the identified candidate drugs, we conducted in vitro bioactivity measurements on the commercially available drugs. The MTT assay was employed to evaluate the effect of candidate drugs on the proliferation of the murine colorectal cell line CT26. The findings indicated that all drugs tested effectively reduced the viability of CT26 cells. Notably, Obatoclax exhibited significant inhibition of CT26 cells at lower concentrations, whereas BAY-87-2243 did not consistently inhibit cell growth. In addition, both ABT-737 and lorlatinib demonstrate inhibitory effects on CT26 cell proliferation at higher concentrations (Fig. 6A). Additionally, a concentration that exhibited no impact on cell proliferation after 48 h was chosen to evaluate its influence on the migration of CT26 cells. The findings demonstrated that Obatoclax and BAY-87-2243 exhibited significant inhibitory effects on the migration of CT26 cells at concentrations as low as 0.01 µM and 0.3 µM, respectively. ABT-737 at a concentration of 10 µM exhibited the most potent inhibitory effects, whereas the remaining drugs did not significantly affect the migration of CT26 cells (Fig. 6B).

### The candidate drug Obatoclax significantly inhibits colorectal liver metastasis

**Fig. 7:**
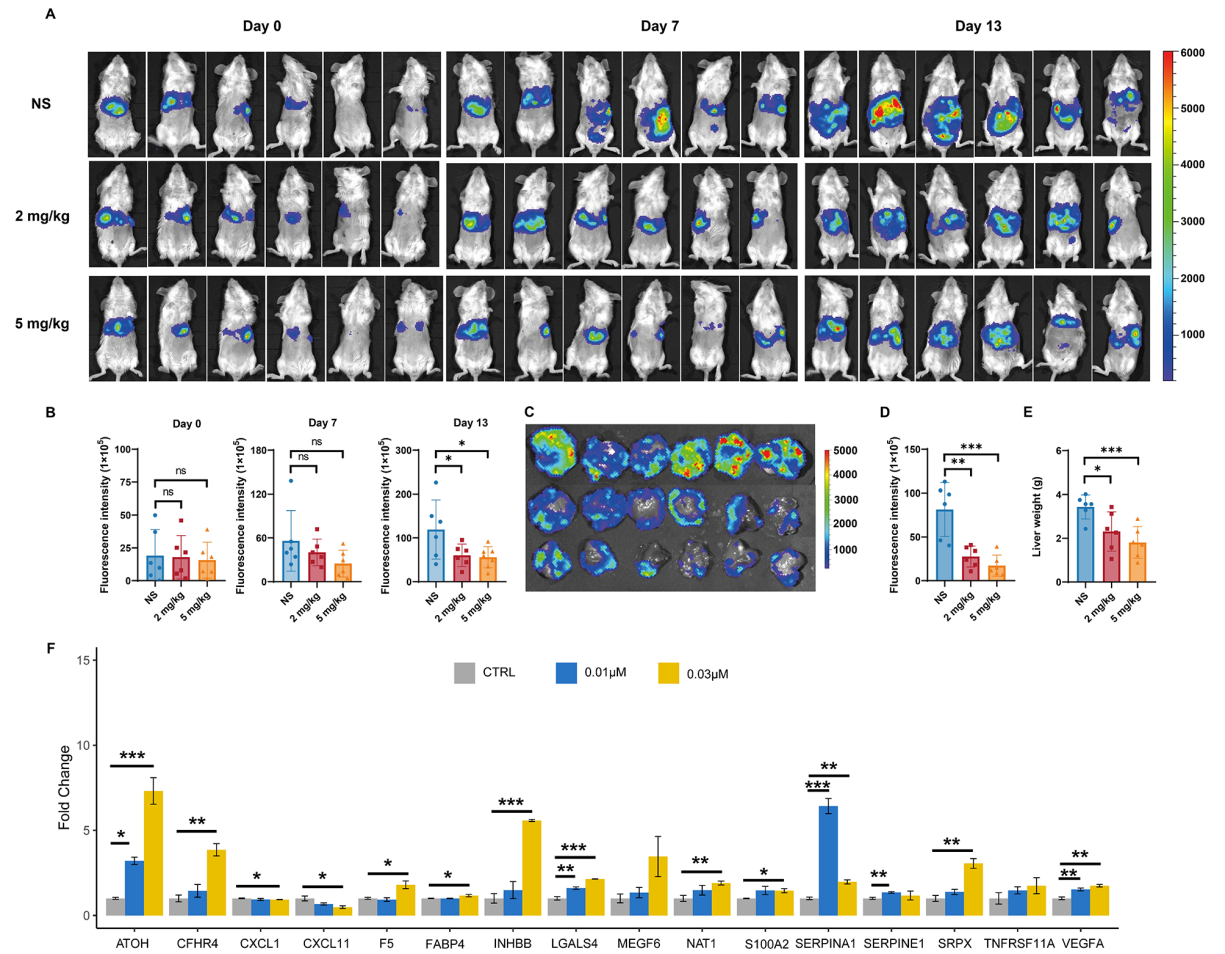
The effects of Obatoclax on the liver metastasis of colorectal cancer in vivo. (**A**) Representative IVIS luciferase in vivo images of mice with CT26-Luc2 cells injected in the spleen and treated with normal saline (NS), 2 mg/kg or 5 mg/kg Obatoclax (*n* = 6). The images obtained on indicated days were quantified with a unified fluorescence scale. (**B**) Summary data of the fluorescence intensity of the mice treated on day 0, 7 and 13. *P < 0.05. (**C**) Images of liver from the tumor bearing mice on day 13. The images obtained were quantified with a unified fluorescence scale. (**D**) Summary data of fluorescence intensity of the liver. **P < 0.01, *** *P* < 0.001. (**E**) Summary data of the liver weight. **P < 0.01, *** *P* < 0.001. (**F**) The fold change (FC) in mRNA expression of MAOS and MAPS signature genes in the CT26 cell line before and after treatment with Obatoclax. FC values were calculated by converting the normalized average log2 values of gene expression levels before and after treatment with Obatoclax at concentrations of 0.01 µM and 0.3 µM

We established a mouse model of colorectal liver metastasis by using CT26-Luc2 cells that express luciferase, allowing for the tracking of tumor metastasis in vivo. Obatoclax, which has a well-defined pharmacodynamic analysis, was chosen for the in vivo experiment from the pool of candidates. Once the model was established, mice were divided into different groups based on their uniform fluorescence levels (Fig. 7A and B). Obatoclax demonstrated significant inhibition of liver metastasis at doses of 2 mg/kg and 5 mg/kg. Following the completion of the treatment, the livers of the mice with tumors were dissected and subjected to IVIS imaging. Obatoclax effectively suppressed liver metastasis in tumor-bearing mice (Fig. 7C and D). The liver weight of Obatoclax-treated mice was significantly lower compared to the control group (Fig. 7E). Obatoclax, the predicted candidate, has demonstrated significant suppression of colorectal liver metastasis, suggesting its potential as a therapeutic drug. We measured the expression levels of MAOS and MAPS signature genes in the CT26 cell line treated with control or Obatoclax at concentrations of 0.01 µM and 0.3 µM. The expression levels of ATOH, CFHR4, CXCL1, F5, LGALS4, SERPINA1, and VEGFA were found to be significantly increased after Obatoclax treatment, as shown in Fig. 7F. This observation supports the hypothesis that the negative correlation between the signature and drug response indicates its potential to decrease the risk of CRLM.

## Discussion

In recent years, researchers have identified novel drug targets and biomarkers through the utilization of next-generation sequencing, leading to enhanced patient outcomes. The availability of extensive transcriptome data, drug sensitivity databases, and computational methods presents a valuable opportunity to understand disease and drug mechanisms and generate novel therapeutic hypotheses [23, 36]. The liver possesses immune tolerance and metabolic activity. Metastasis to the liver necessitates intricate biological mechanisms, as proposed by the “seed and soil” hypothesis [37]. Liver metastases showed decreased epithelial-mesenchymal transition and increased activity in the MYC target and DNA-repair pathways, according to the analysis of differentially expressed genes [8, 38]. In addition, we observed several metabolic pathways, such as xenobiotic, bile acid, heme metabolism, and cholesterol homeostasis, that were enriched in liver metastases (Fig. 1C and D). Recent studies indicate that metastatic cells may exploit altered metabolism to evade immune detection [39, 40]. Given the distinctive molecular properties of CRLM and the limited therapeutic alternatives, it is crucial to customize specialized treatment for these individuals. This study represents the initial endeavor to combine prognostic prediction for CRLM with computational drug repositioning, aiming to facilitate treatment development.

In order to predict the prognosis and categorize patients with colorectal liver metastasis (CRLM), we developed two liver metastasis-related signatures (MAOS and MAPS) using survival data on overall survival (OS) and progression-free interval (PFI). The occurrence of overall survival (OS) is frequently used as a primary outcome measure in clinical studies. However, the absence of specific information regarding the cause of death can potentially weaken the validity and reliability of these studies. PFI is a survival endpoint that takes into account disease progression, locoregional recurrence, and distant metastasis, making it more relevant to metastasis-related outcomes [29]. Hence, we integrated PFI into our prognostic model to enhance the screening of candidate genes linked to metastasis. Both MAOS and MAPS studies identified multiple genes that have been reported to have a strong association with colorectal cancer. LGALS4, a lectin galactoside-binding soluble 4, exhibits specific expression in the colon and plays a role in the invasion of cancer cells [41]. MEGF6, a protein with multiple epidermal growth factor-like domains, has been identified as an oncogene that facilitates cell proliferation, suppresses apoptosis, and enhances cell migration through the TGFβ/SMAD signaling pathway-mediated EMT [42].

MAOS encompassed genes associated with metastasis-promoting pathways, including epithelial mesenchymal transition and lipid metabolism. CXCL1 is upregulated in liver tissue, which attracts myeloid-derived suppressor cells that express CXCR2. This ultimately results in the creation of a premetastatic niche for liver metastases [43, 44]. The overexpression of FABP4 facilitates the transportation of fatty acids, resulting in enhanced energy and lipid metabolism. Additionally, it activated the AKT pathway and epithelial-mesenchymal transition, promoting the movement and infiltration of colon cancer cells [45, 46]. The autophagy-related gene SERPINA1 has been demonstrated to affect the invasive and metastatic capabilities in colorectal cancer [47, 48].

The remaining six genes within the MAPS (CFHR4, F5, S100A2, SRPX, SERPINE1, and VEGFA) have demonstrated their independent value as prognostic biomarkers in various solid tumors. The F5 expression has been associated with tumor aggressiveness and has been identified as an immunological marker for the cancer-inflammation-thrombosis circuit in breast cancer [49]. S100A2 protein overexpression has been identified as a prognostic indicator for patients diagnosed with stage II and III colorectal cancer [50]. The study observed a significant increase in the expression of the SRPX gene in cancer-associated fibroblasts of high-grade serous carcinoma, clear cell carcinoma, and ovarian carcinoma [51]. Vascular endothelial growth factor (VEGFA) was a major mediator of angiogenesis process in CRC [52]. Moreover, the secretion of VEGFA by colorectal cancer cells induces tumor-associated macrophages to produce CXCL1 and attract MDSCs, leading to the formation of a premetastatic niche that facilitates the development of liver metastases [43]. The significant correlation between high TNM stages and elevated MAOS/MAPS scores suggests that our signature genes are associated with disease progression (Table 1). This correlation further extends to lymph node metastases, highlighting the MAOS/MAPS scores’ potential in distinguishing patients at elevated risk of lymph node involvement. Higher MAOS/MAPS scores may indicate a higher risk of distant spread, guiding decisions on treatment strategies and surveillance. Additionally, the relationship between MAOS/MAPS scores and microsatellite instability (MSI) underscores the utility of these scores in pinpointing patients with specific molecular profiles, potentially influencing personalized treatment decisions based on MSI status. Both MAOS and MAPS exhibited superior performance compared to other prognostic signatures in both the training and testing sets. Despite using overall survival (OS) as the survival endpoint in the training set, MAPS demonstrated superior performance compared to MAOS, suggesting that the biomarker has good generalizability. MAOS outperformed MAPS slightly in the external testing dataset. MAOS and MAPS can serve as biomarkers for targeted CRLM treatment, in addition to their prognostic value.

We further screened candidate drugs for CRLM using drug response deep learning models trained with CCLE and GDSC drug sensitivity datasets. The candidate drugs were assessed using MTT and Transwell assays in vitro, and all demonstrated inhibitory effects on the proliferation of CT26 cells. Obatoclax, BAY-87-2243, and ABT-737 were found to effectively inhibit the cell migration of CT26. Obatoclax demonstrated a significant inhibitory effect on liver metastasis in tumor-bearing mice. Obatoclax is a pan-BCL-2 family inhibitor targeting mitochondrial apoptotic pathways. It was approved by the U.S. Food and Drug Administration (FDA) to treat chronic lymphocytic leukemia [53, 54]. Apoptosis has been a promising target for anticancer therapy in the past decades and BCL-2 proteins and their interactions can induce apoptosis through intrinsic pathways [55, 56]. Recent discoveries in the study of apoptosis and the tumor microenvironment have revealed that BCL-2 proteins are also important mediators of metabolic pathways [40, 57–59]. Previous studies have also shown that Obatoclax can inhibit the migration and proliferation of colorectal cancer cells [60, 61], and increase the chemosensitivity of colon cancer cells to fluorouracil by reducing hypoxia-inducible factor (HIF)-1 transcriptional activity [62].

Recent research using scRNA-seq indicates that the tumor heterogeneity contributes to drug resistance and treatment failure in cancer therapy. However, the accuracy of drug response prediction is restricted by the limited size and considerable cost of available single cell drug perturbation data. Currently, most drug response models utilize the high-throughput drug screening database on cancer cell lines to make inferences on drug responses. Since the cancer cell line gene expression profiles and most tumor samples were analyzed by bulk RNA-seq, which captures an averaged estimation across tumor cell types, specialized methods are needed to utilize large-scale drug screens of CCLs to predict drug response at the cellular level.

The inherent benefit of employing a deep learning-based framework for drug response prediction is a subject of considerable interest within the field of bioinformatics. Deep learning models can effectively handle diverse scRNA-seq data and accurately predict cellular drug sensitivity labels due to their flexibility and ability to learn complex relationships [63]. For example, scDEAL is a deep transfer learning method based on neural network architecture, which combines bulk and scRNA-seq data to predict cancer drug response [64]. SCAD implemented an adversarial learning approach by training a domain discriminator to address cross-domain bias between bulk and scRNA-seq datasets [65]. However, a major limiting factor against predicting drug response in heterogeneous tumor samples is insufficient training power due to the lack of public benchmark data at the single-cell level.

In this study, we attempted to address the above-mentioned challenge by integrating deep learning methods and signature-based methods to explore their ability to predict drug response. Instead of relying on detection of shared expression patterns between bulk and scRNA-seq data, the identification of biomarkers depends only on bulk RNA-seq and bulk sample labels. Drug response can be predicted based on the hypothesis that drug gene expression patterns, which have the potential to counteract the gene expression signature associated with the malignant or high-risk phenotype, are more likely to have therapeutic effects [4]. Using similar approach, the Beyondcell method successfully identified distinct drug response subpopulations before and after bortezomib treatment in a breast cancer single-cell dataset [5].

The deep learning drug response prediction model offers several advantages. The reason for its effectiveness lies in its integration of pathway activity estimation and drug descriptors as features [28]. One benefit is that drugs’ structural characteristics may be used as explanatory variables to predict pharmaceuticals that are absent from the training set. The interpretability of drug response predictions was enhanced by using pathway enrichment scores rather than gene expression levels. Our application of Precily model on the CRLM scRNA-seq dataset illustrated that the Precily model, initially trained on bulk cancer cell line drug perturbation data, can be effectively extended for predicting drug responses at the single-cell level. However, a significant challenge in accurately predicting drug responses at the single-cell level is the insufficient training capacity, primarily due to the absence of publicly benchmarked data, which hinders the ability to make precise predictions. This aspect of data availability and its impact on predictive accuracy is an important consideration for advancing the field of personalized medicine.

The analysis of signaling pathways associated with MAOS and MAPS scores reveals that the apoptotic pathway is more prevalent in low-risk CRLM patients. This finding aligns with the targeting pathway of Obatoclax and ABT-737, providing evidence for the effectiveness and accuracy of Precily’s prediction. Phosphorylation and N-methyl-D-aspartate receptor-related signaling pathways were found to be enriched in drug-resistant, high-risk patients with CRLM (Supplementary Figure S9A and B). The enriched pathways showed significant negative correlations with the drug response signatures of Obatoclax and ABT-737. Additionally, they exhibited significant correlations with genes that are highly expressed in myeloid and stromal cells among the MAOS and MAPS signature genes (Supplementary Figure S9C and D). This finding indicates that the drugs we tested have the potential to modulate intercellular communication in the tumor microenvironment of CRLM by influencing specific signaling pathways, ultimately leading to the induction of tumor cell apoptosis. This approach allows for a broader understanding of how specific pathways are dysregulated in CRLM patients.

In summary, we have developed two signatures that can be used to stratify high-risk CRLM based on their gene expression characteristics. We employed deep learning-based drug response prediction models to screen candidate drugs for high-risk CRLMs. We subsequently validated our findings through in vitro and in vivo experiments. Our findings may provide new insights into the targeted treatment of CRLM. Further validation is required to determine the prognostic value of MAOS and MAPS. To validate the accuracy of current prognostic models, we utilize OS data as PFI information may not be available in all datasets. Despite its limitations, this study contributes to potential advancements in the precise treatment of colorectal liver metastases (CRLM).

## Conclusions

In conclusion, our study has successfully established two gene signatures, namely MAOS and MAPS, which can be utilized to predict the prognosis of CRC patients with liver metastases. The MAOS and MAPS are highly therapeutically relevant in stratifying patients with CRLM into high- and low-risk groups. In contrast to traditional drug discovery strategies based on a specific drug target, the study applied an interpretable deep learning model and drug sensitivity databases to predict drug responses and screen potential therapeutic drugs by integrating pathway activity estimation and drug descriptors. Finally, our study identified a BCL-2 inhibitor Obatoclax through wet-lab in vitro assays and a colorectal liver metastasis model. This study has provided biomarkers for colorectal liver metastasis and has also contributed to the development of novel drug discovery strategies for precision oncology.

### Electronic supplementary material

Below is the link to the electronic supplementary material.

Supplementary Material 1

Supplementary Material 2

## Data Availability

All datasets utilized in this study are publicly available. Microarray gene expression and related clinical data were sourced from the Gene Expression Omnibus (GEO). RNA-seq gene expression and associated clinical data from the TCGA project were acquired via the Genomic Data Commons (accessible at https://gdc.cancer.gov/access-data/). Molecular and dependency data for cancer cell lines were obtained from the DepMap portal (https://depmap.org/portal/). Drug response data for cancer cell lines were collected from both CTRPv2 (https://portals.broadinstitute.org/ctrp), Genomics of Drug Sensitivity in Cancer (GDSC2, https://cog.sanger.ac.uk/cancerrxgene/), and PRISM (https://depmap.org/portal/prism/).

## Abbreviations

CRLM: Colorectal cancer with liver metastasis
MSI: microsatellite instability
CCLE: Cancer Cell Line Encyclopedia
CTRP: Cancer Therapeutics Response Portal
GDSC: Genomics of Drug Sensitivity in Cancer
PRISM: Profiling Relative Inhibition Simultaneously in Mixtures
DNN: deep neural network
DEGs: differentially expressed genes
NC: normal colon
LM: liver metastasis
PT: primary colorectal tumor
TCGA: The Cancer Genome Atlas
GEO: Gene Expression Omnibus dataset
IC_50_: half-maximal inhibitory concentration
MAOS: metastasis associated overall survival signature
MAPS: metastasis associated progression signature
