# Supplementary material for: An integrated framework for prognosis prediction and drug response modeling in colorectal liver metastasis drug discovery
Source: J Transl Med. 2024 Mar 30;22:321. doi: 10.1186/s12967-024-05127-5 (PMC10981831; doi:10.1186/s12967-024-05127-5)
Supplement: Supplementary file 1 — Supplementary Material 1 [file 12967_2024_5127_MOESM1_ESM.docx]

**Supplementary Information**

**Integrated Prognosis and Deep Learning Drug Response Models for Drug Discovery in Colorectal Liver Metastasis**

Xiuman Zhou et al.

**Figure S1.** Batch Effect Analysis of the Combined Gene Expression Cohort (Datasets: GSE68468, GSE41568, and GSE81558). (A) Principal Component Analysis (PCA) plot showing sample distribution before batch effect correction, labeled by sampling types: normal colon (NC), primary tumor (PT), and liver metastasis (LM). (B) PCA plot showing sample distribution after batch effect correction. (C) PCA plot showing sample distribution before batch effect correction, labeled by source datasets. (D) PCA plot showing sample distribution after batch effect correction, labeled by source datasets.


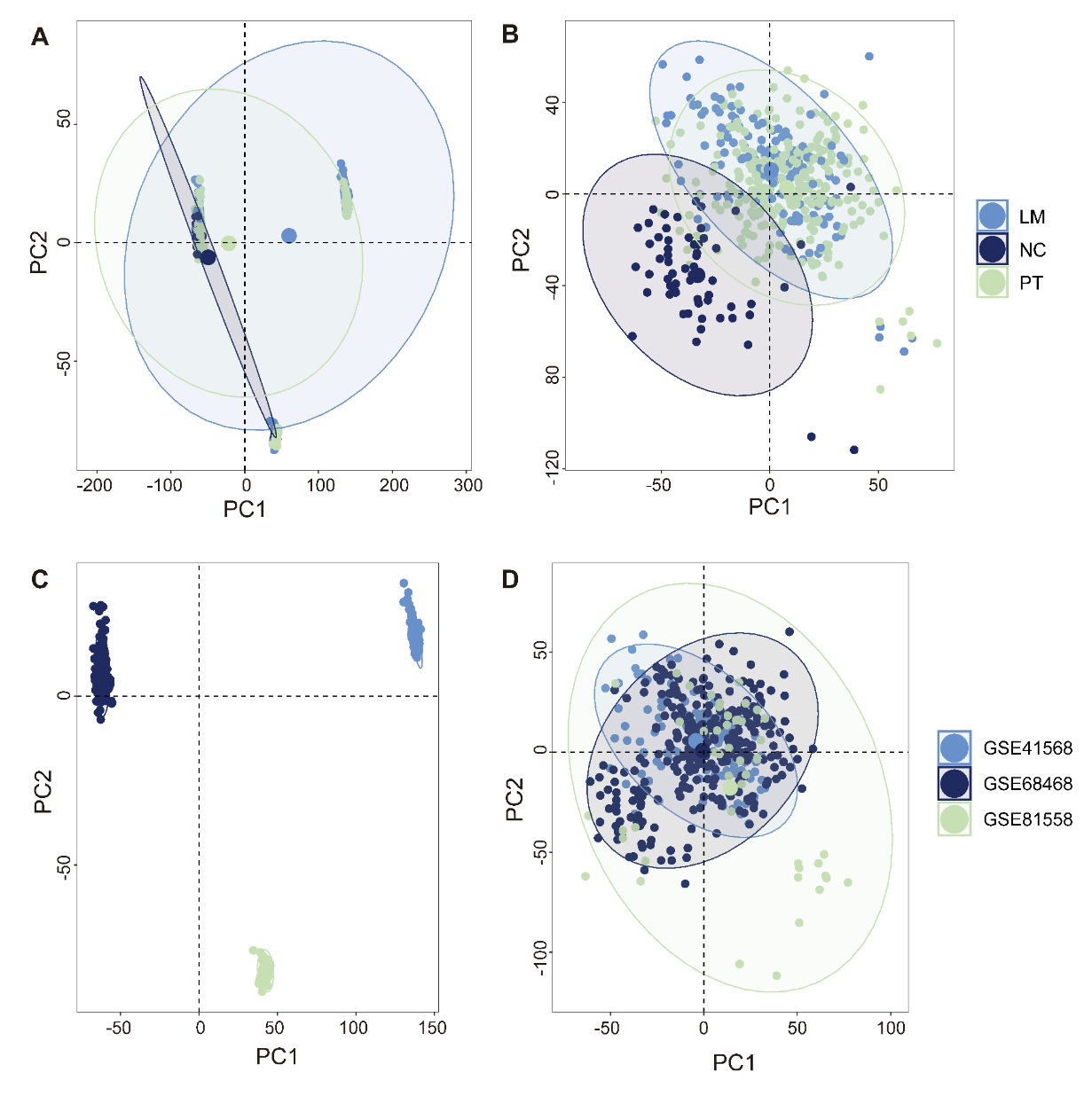


**Table S1.** The platform, number of samples and sampling types of the GEO datasets included in this study.

| Dataset | Platform | Samples | Peritumor | Primary Tumor | Liver Metastases |
| --- | --- | --- | --- | --- | --- |
| GSE68468 | GPL96 | 310 | 55 | 195 | 47 |
| GSE41568 | GPL570 | 118 | - | 39 | 79 |
| GSE81558 | GPL15207 | 51 | 9 | 23 | 19 |
| GSE39582 | GPL570 | 585 | - | 585 | - |
| GSE17536 | GPL570 | 177 | - | 177 | - |

**Table S2.** Univariate Cox regression analysis of MAOS signature genes in TCGA.

| Gene | HR | Lower (95%CI) | Upper (95%CI) | *P*-value |
| --- | --- | --- | --- | --- |
| ATOH1 | 0.893 | 0.828 | 0.962 | 0.003 |
| CXCL1 | 0.828 | 0.689 | 0.995 | 0.044 |
| FABP4 | 1.196 | 1.039 | 1.376 | 0.013 |
| INHBB | 1.106 | 1.014 | 1.206 | 0.023 |
| LGALS4 | 0.827 | 0.727 | 0.942 | 0.004 |
| MEGF6 | 1.079 | 1.014 | 1.149 | 0.017 |
| NAT1 | 0.708 | 0.587 | 0.855 | 0.000 |
| SCGB2A1 | 0.753 | 0.588 | 0.964 | 0.024 |
| SERPINA1 | 0.831 | 0.721 | 0.958 | 0.011 |
| TNFRSF11A | 0.883 | 0.795 | 0.979 | 0.019 |

**Table S3.** Univariate Cox regression analysis of MAPS signature genes in TCGA.

| Gene | HR | Lower (95%CI) | Upper (95%CI) | *P*-value |
| --- | --- | --- | --- | --- |
| CFHR4 | 1.021 | 1.008 | 1.034 | 0.002 |
| CXCL11 | 0.776 | 0.669 | 0.901 | 0.001 |
| F5 | 1.035 | 1.007 | 1.063 | 0.015 |
| INHBB | 1.169 | 1.084 | 1.261 | 0.000 |
| LGALS4 | 0.799 | 0.709 | 0.900 | 0.000 |
| MEGF6 | 1.072 | 1.012 | 1.137 | 0.019 |
| NAT1 | 0.776 | 0.656 | 0.917 | 0.003 |
| S100A2 | 1.073 | 1.023 | 1.125 | 0.004 |
| SERPINE1 | 1.256 | 1.080 | 1.460 | 0.003 |
| SRPX | 1.330 | 1.085 | 1.630 | 0.006 |
| VEGFA | 1.169 | 1.035 | 1.319 | 0.012 |

**Table S4.** Detailed information of MAOS and MAPS signature genes and the corresponding standardized coefficients in the prognostic models.

| List of 10 genes in MAOS | | | List of 11 genes in MAPS | | |
| --- | --- | --- | --- | --- | --- |
| Symbol | Description | Coefficient | Symbol | Description | Coefficient |
| NAT1 | Polymorphic enzyme N-acetyltransferase 1 | -0.182 | NAT1 | Polymorphic enzyme N-acetyltransferase 1 | -0.015 |
| MEGF6 | Multiple epidermal growth factor-like domains protein 6 | 0.026 | MEGF6 | Multiple epidermal growth factor-like domains protein 6 | 0.011 |
| INHBB | Inhibin subunit beta | 0.026 | INHBB | Inhibin subunit beta | 0.056 |
| LGALS4 | Lectin galactoside-binding soluble 4 | -0.027 | LGALS4 | Lectin galactoside-binding soluble 4 | -0.143 |
| SCGB2A1 | Secretoglobin Family 2A Member 1) | -0.01 | SERPINE1 | Serpin Family E Member 1 | 0.052 |
| FABP4 | Fatty Acid Binding Protein 4 | 0.075 | VEGFA | Vascular Endothelial Growth Factor A) | 0.06 |
| ATOH1 | Atonal BHLH Transcription Factor 1 | -0.032 | SRPX | Sushi Repeat Containing Protein X-Linked | 0.071 |
| TNFRSF11A | TNF Receptor Superfamily Member 11a | -0.001 | F5 | Coagulation Factor V | 0.005 |
| CXCL1 | C-X-C Motif Chemokine Ligand 1 | -0.037 | S100A2 | S100 Calcium Binding Protein A2 | 0.039 |
| SERPINA1 | Serpin Family A Member 1 | -0.024 | CXCL11 | C-X-C motif chemokine ligand 11 | -0.231 |
|  |  |  | CFHR4 | Complement Factor H Related 4 | 0.01 |

**Figure S2.** Kaplan-Meier survival analysis of the 10 MAOS signature genes in the TCGA COAD dataset, with "High" and "Low" defined by median MAOS scores.
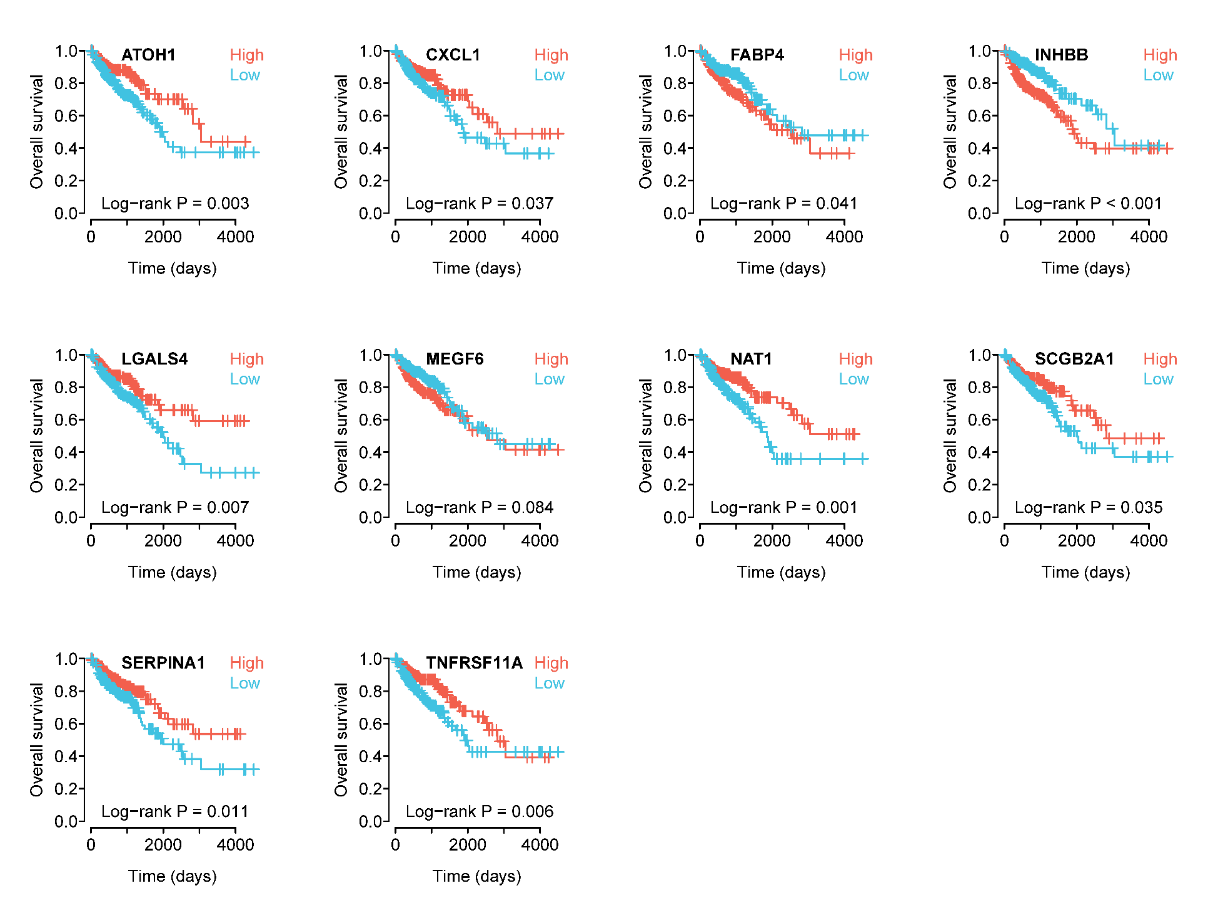


**Figure S3.** Kaplan-Meier survival analysis of the 10 MAPS signature genes in the TCGA COAD dataset, with "High" and "Low" defined by median MAPS scores.
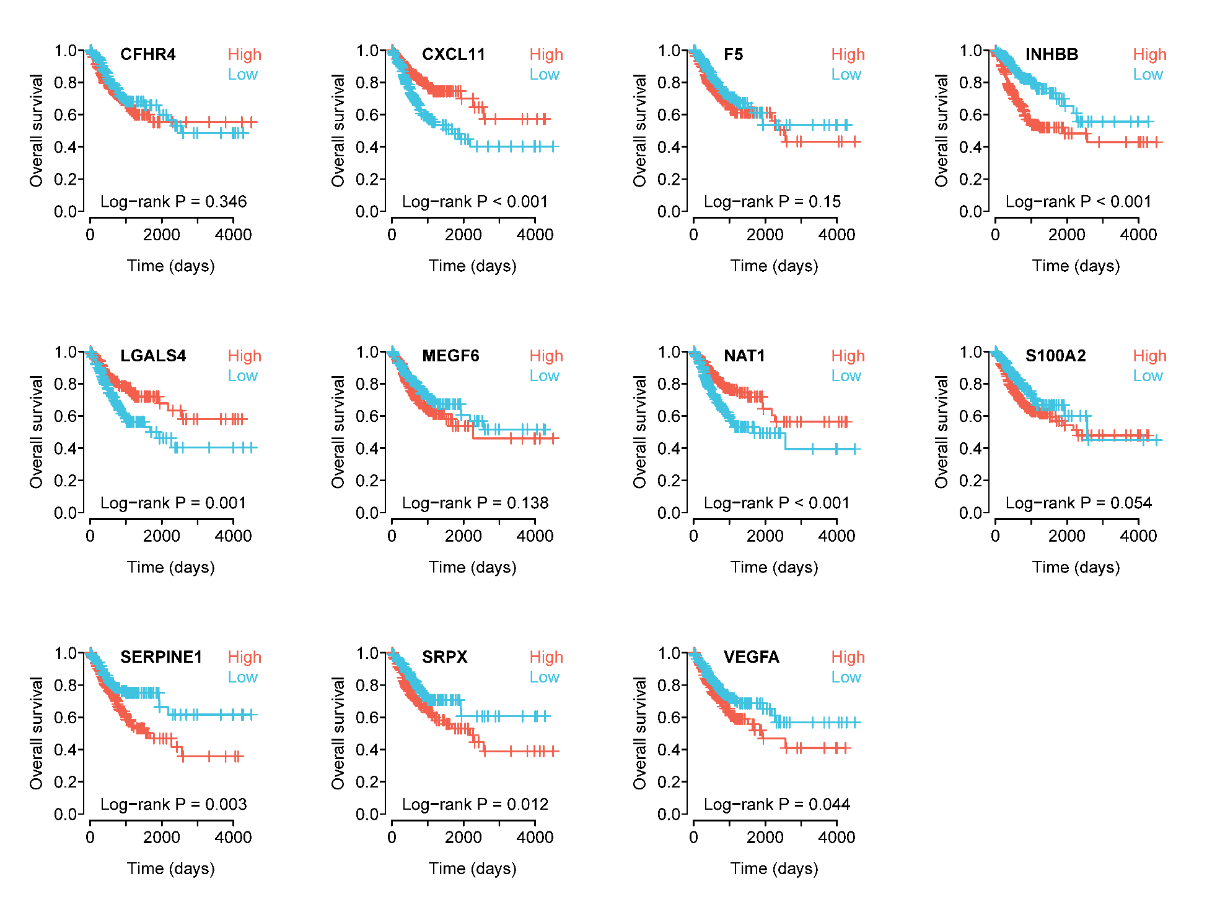


**Figure S4.** Bubble plot of the average and percent expression of MAOS and MAPS signature genes across various cell subtypes of CRLM, as observed in the single-cell RNA sequencing dataset GSE225857.

**
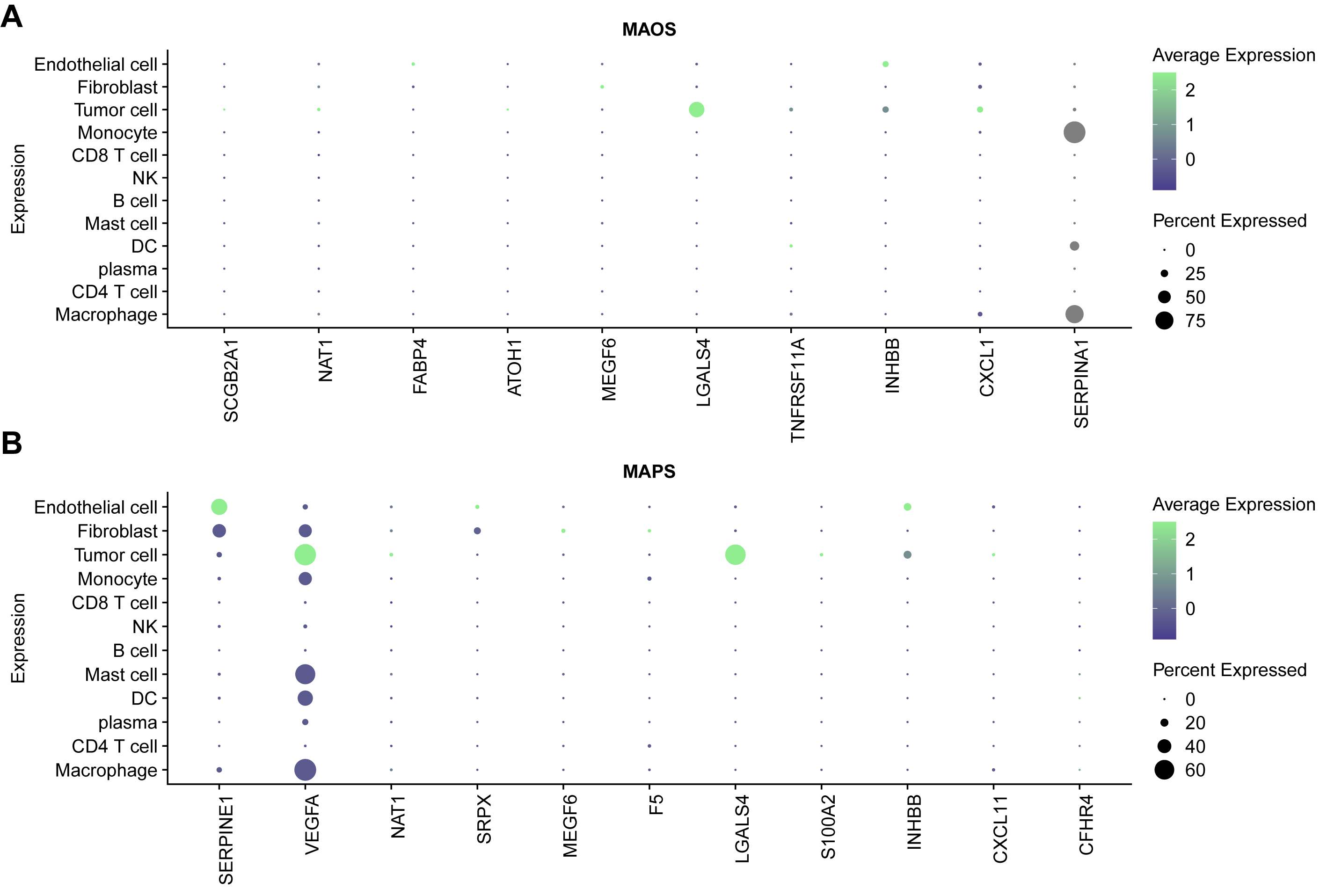
**

**Figure S5** Tumor heterogeneity in colorectal cancer liver metastasis (CRLM) and its impact on drug response. (A) a bar plot of single-cell data from seven CRLM patient samples. (B) The overall predicted IC_50_ values combining all CRLM samples as well as predicted IC_50_ values in each CRLM sample, among approved and candidate drugs for CRLM. (C) Box plots of the comparison of predicted IC_50_ of approved and candidate CRLM drugs among different cell types. Lower IC_50_ values imply greater drug sensitivity.


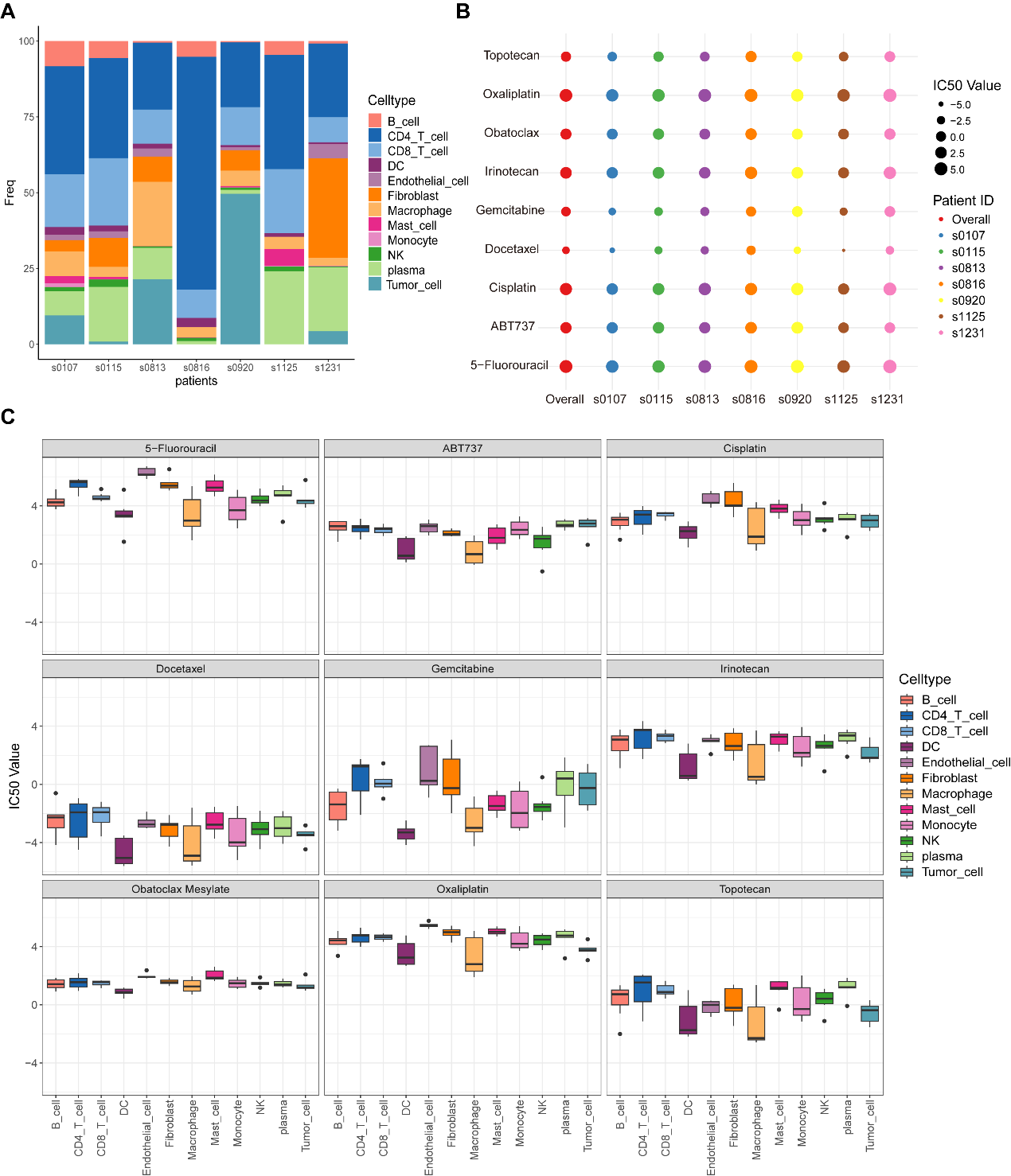


**Figure S6.** Bubble plot of significant different pathways (Welch's t-test *P* < 0.01) in 12 cell types between CRLM patients with high- and low-MAOS scores. CRLM patients were categorized into high and low risk groups based on the median values of MAOS scores.

**
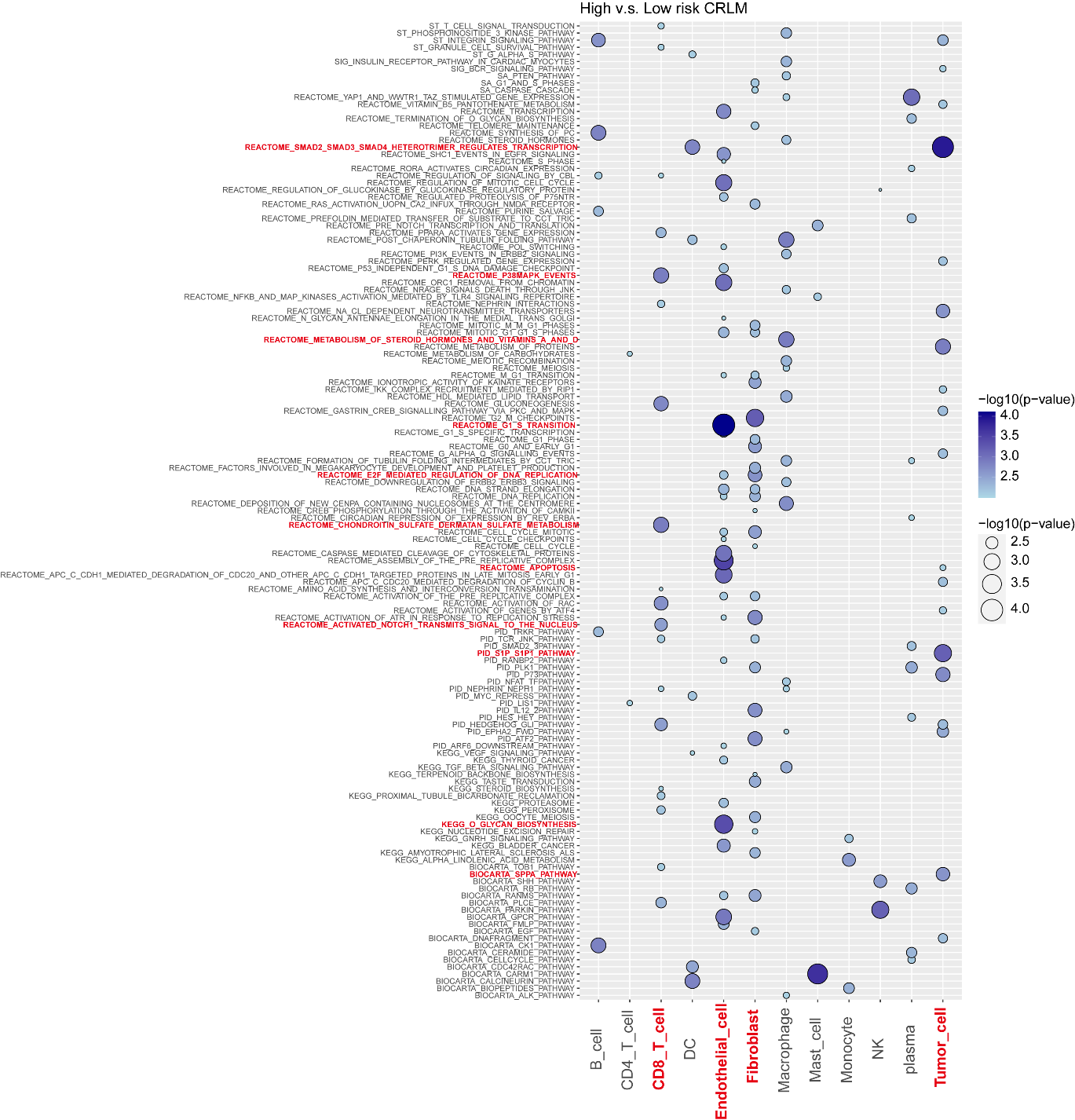
**

**Figure S7.** Bubble plot of significant different pathways (Welch's t-test *P* < 0.001) in 10 cell types between CRLM patients with high- and low-MAPS scores. CRLM patients were categorized into high and low risk groups based on the median values of MAPS scores.


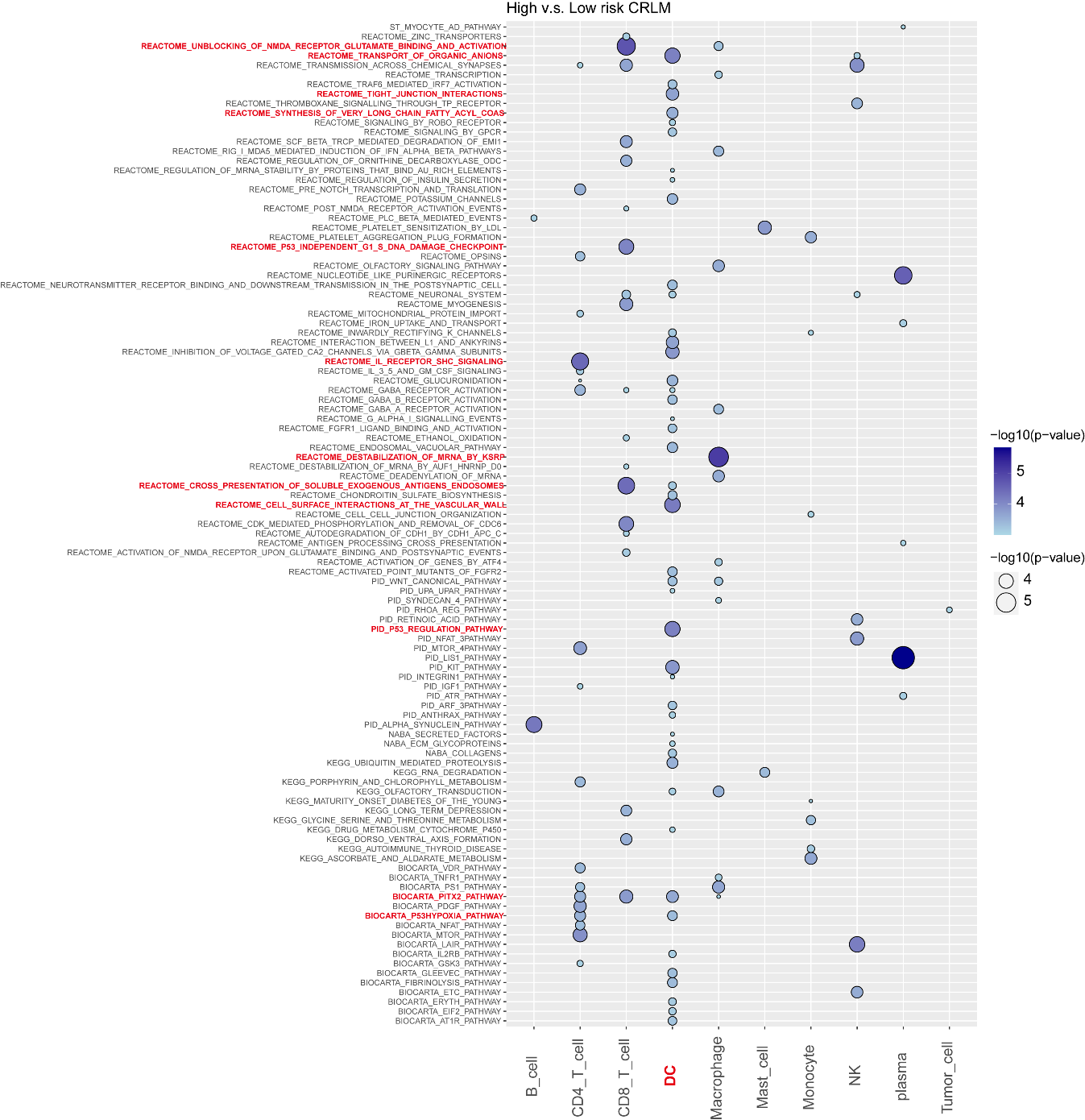


**Figure S8.** Identification of cell-type-specific drugs for high risk CRLM patients with CRLM scRNA-seq dataset GSE225857. (A) Bubble plot of drugs with significant different IC_50_ values (Welch's t-test *P* < 0.01) in 11 cell types between CRLM patients with high- and low-MAOS scores. (B) Bubble plot of drugs with significant different IC_50_ values (Welch's t-test *P* < 0.005) in 10 cell types between CRLM patients with high- and low-MAPS scores. CRLM patients were categorized into high and low risk groups based on the median values of MAOS and MAPS scores, respectively.


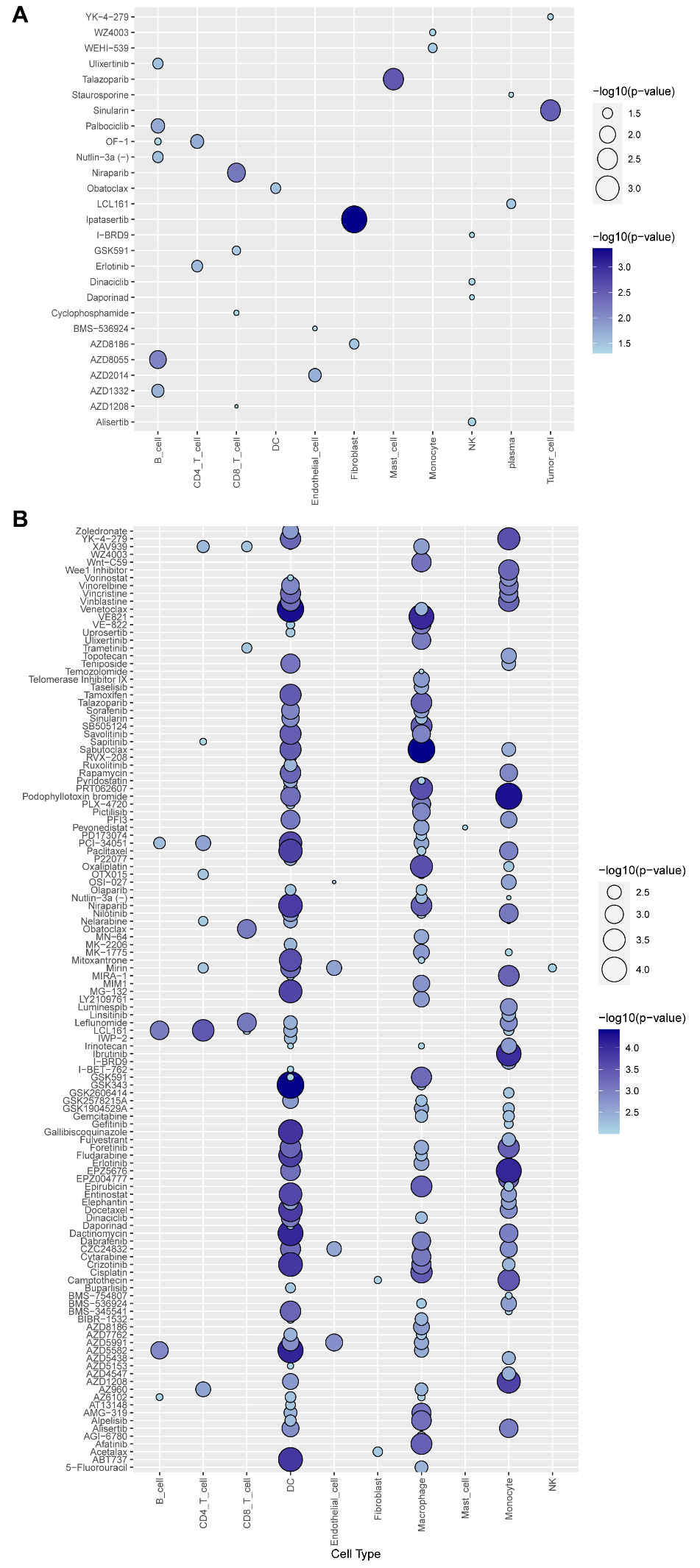


**Figure S9.** Identification of signaling pathways related to drug response in the CRLM patients. (A) Heatmap of significant signaling pathways correlated with MAOS. *P*-values were obtained from the two-sided Wilcoxon rank-sum test between high- and low-MAOS groups. (B) Heatmap of significant signaling pathways correlated with MAPS. *P*-values were obtained from the two-sided Wilcoxon rank-sum test between high- and low-MAPS groups. (C) Heatmap of the correlation between the predicted IC50 of candidate drugs and enriched signaling pathway scores in the CRLM cohort. *P*-values were obtained from Spearman’s correlation test. (D) Bubble plot of correlation between the enriched signaling pathway scores and signature genes of MAOS and MAPS *, *P* < 0.05, **, *P* < 0.01, ***, *P* < 0.001.

**
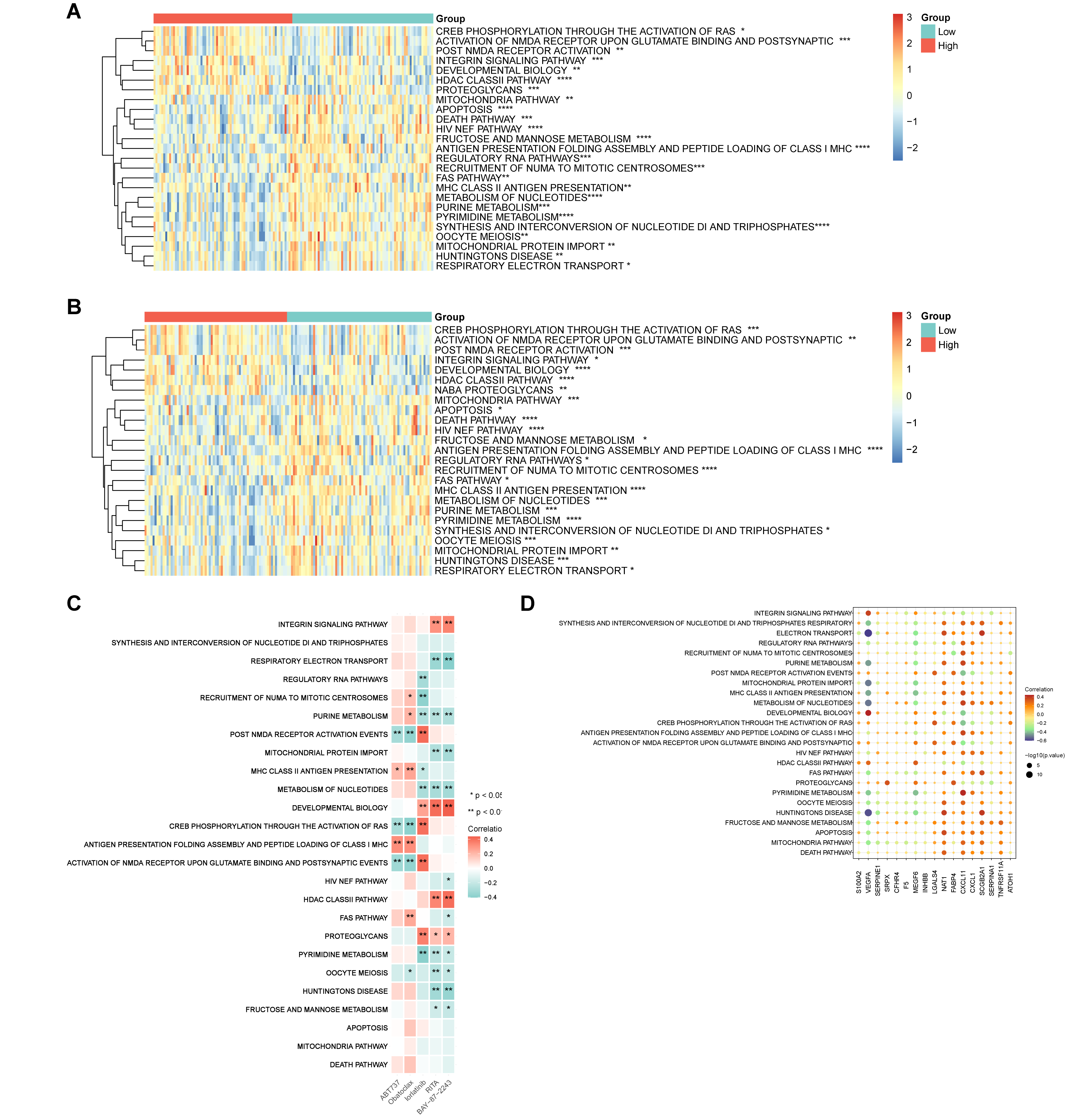
**
