# Supplementary material for: An integrated framework for prognosis prediction and drug response modeling in colorectal liver metastasis drug discovery
Source: J Transl Med. 2024 Mar 30;22:321. doi: 10.1186/s12967-024-05127-5 (PMC10981831; doi:10.1186/s12967-024-05127-5)
Supplement: Supplementary file 2 — Supplementary Material 2 [file 12967_2024_5127_MOESM2_ESM.docx]

**Response letter**

Mar. 21th, 2024

**Dear Editors and Reviewers,**

Thank you very much for your valuable comments and suggestions on the manuscript (**JTRM-D-23-08539R1**) entitled “**An Integrated Framework for Prognosis Prediction and Drug Response Modeling in Colorectal Liver Metastasis Drug Discovery**”. We have thoroughly revised our manuscript according to the comments from the reviewers point by point. Other mistakes were also carefully reviewed and corrected. The revised manuscript indicate all the changes with blue highlighting.

I eagerly await your favorable reply and am available for any further clarifications or revisions that may be required.

Sincerely,

Xinghua Sui, Ph.D.

School of Pharmaceutical Sciences (Shenzhen)

Shenzhen Campus of Sun Yat-sen University

Shenzhen 518107, P. R. China

E-mail: [suixingh@mail.sysu.edu.cn](mailto:suixingh@mail.sysu.edu.cn)

**Below are point and point response to the comments from Reviewers (reviewer's comments in *italic*).**

***Reviewer #2:***

*GENERAL COMMENTS: I thank the authors very much for their comprehensive response to my previous comments. Overall, my concerns have been adequately addressed, and I am satisfied with the current version of the manuscript.*

**Response:** Thank you for your constructive comments regarding our study.

*REQUESTED REVISIONS:*

*However, I would like to highlight the following points for further consideration:*

*The authors' response regarding* *the contributions of the identified clinical characteristics to the Integrated Prognosis and Drug Discovery model should be incorporated into the Discussion section for a more comprehensive understanding.*

**Response:** Thank you for your constructive suggestion. We have revised the Discussion section to succinctly articulate the contributions of the identified clinical characteristics to the Integrated Prognosis and Drug Discovery model as follows (line 489 to 497 on page 24):

“The significant correlation between high TNM stages and elevated MAOS/MAPS scores suggests that our signature genes are associated with disease progression (Table 1). This correlation further extends to lymph node metastases, highlighting the MAOS/MAPS scores' potential in distinguishing patients at elevated risk of lymph node involvement. Higher MAOS/MAPS scores may indicate a higher risk of distant spread, guiding decisions on treatment strategies and surveillance. Additionally, the relationship between MAOS/MAPS scores and microsatellite instability (MSI) underscores the utility of these scores in pinpointing patients with specific molecular profiles, potentially influencing personalized treatment decisions based on MSI status. “

*The authors mentioned that they "did not standardize the gene expression values before including them in the calculation of the risk scores." This should be acknowledged as a limitation in Discussion section. Generally, either the original gene expression values or the estimated coefficients should be scaled or standardized before being used in subsequent analyses. This point is crucial for the integrity and reliability of the study's findings.*

**Response:** We sincerely appreciate the reviewer for pointing out this critical aspect. We apologize for any previous misunderstanding. To clarify, we would like to state that our gene expression of TCGA-COAD cohort was indeed standardized using the z-score method before included into the risk score calculations. Our initial response misunderstood the reviewer's comment, mistakenly thinking the inquiry was about whether we had applied additional transformations to the expression values. We understand that standardizing expression data is a fundamental and crucial step in constructing prognostic models to ensure the generalizability of the prognostic model. It ensures that variations in gene expression levels or coefficient magnitudes across different samples do not bias the results, allowing for accurate and meaningful comparisons across the study cohort. We rephrased the text in the revised manuscript (line 151 to 154 on page 9) and revised caption of Table S4.

“The liver metastasis associated overall survival signature (MAOS) and metastasis associated progression signature (MAPS) were calculated for each patient by summing the products of all signature genes’ regression coefficients multiplied by their corresponding z-score standardized expression values.”

*A minor issue to address is the accurate numbering of tables and figures in the main manuscript and supplementary materials. For instance, what is currently labeled as* *'Figure S2' (the seond Figure S2) should be corrected to 'Figure S3'.*

**Response:** We appreciate your careful review of our manuscript. We revised the figure legend of the second 'Figure S2' to 'Figure S3'. We have carefully checked the rest of the manuscript to ensure that all tables and figures are correctly numbered and correspond accurately to their citations in the text. We apologize for any confusion this may have caused and appreciate your help in improving the quality of our manuscript.
